# Can China achieve a one-third reduction in premature mortality from non-communicable diseases by 2030?

**DOI:** 10.1186/s12916-017-0894-5

**Published:** 2017-07-11

**Authors:** Yichong Li, Xinying Zeng, Jiangmei Liu, Yunning Liu, Shiwei Liu, Peng Yin, Jinlei Qi, Zhenping Zhao, Shicheng Yu, Yuehua Hu, Guangxue He, Alan D. Lopez, George F. Gao, Linhong Wang, Maigeng Zhou

**Affiliations:** 1 National Center for Chronic and Non-communicable Disease Control and Prevention, Chinese Center for Disease Control and Prevention, Nanwei Road 27, Xicheng District, Beijing, China; 2 Chinese Center for Disease Control and Prevention, Beijing, China; 3 Melbourne School of Population and Global Health, University of Melbourne, Melbourne, Australia

**Keywords:** Chronic disease, Mortality, Premature, Forecasting, Risk factors

## Abstract

**Background:**

The United Nation’s Sustainable Development Goals for 2030 include reducing premature mortality from non-communicable diseases (NCDs) by one third. To assess the feasibility of this goal in China, we projected premature mortality in 2030 of NCDs under different risk factor reduction scenarios.

**Methods:**

We used China results from the Global Burden of Disease Study 2013 as empirical data for projections. Deaths between 1990 and 2013 for cardiovascular disease (CVD), diabetes, chronic respiratory disease, cancer, and other NCDs were extracted, along with population numbers. We disaggregated deaths into parts attributable and unattributable to high systolic blood pressure (SBP), smoking, high body mass index (BMI), high total cholesterol, physical inactivity, and high fasting glucose. Risk factor exposure and deaths by NCD category were projected to 2030. Eight simulated scenarios were also constructed to explore how premature mortality will be affected if the World Health Organization’s targets for risk factors reduction are achieved by 2030.

**Results:**

If current trends for each risk factor continued to 2030, the total premature deaths from NCDs would increase from 3.11 million to 3.52 million, but the premature mortality rate would decrease by 13.1%. In the combined scenario in which all risk factor reduction targets are achieved, nearly one million deaths among persons 30 to 70 years old due to NCDs would be avoided, and the one-third reduction goal would be achieved for all NCDs combined. More specifically, the goal would be achieved for CVD and chronic respiratory diseases, but not for cancer and diabetes. Reduction in the prevalence of high SBP, smoking, and high BMI played an important role in achieving the goals.

**Conclusions:**

Reaching the goal of a one-third reduction in premature mortality from NCDs is possible by 2030 if certain targets for risk factor intervention are reached, but more efforts are required to achieve risk factor reduction.

**Electronic supplementary material:**

The online version of this article (doi:10.1186/s12916-017-0894-5) contains supplementary material, which is available to authorized users.

## Background

China has experienced tremendous socioeconomic changes in the past 30 years, during which time rapid economic development substantially influenced the diets and lifestyles of the Chinese population. Meanwhile, a remarkable epidemiological and demographic transition has markedly changed the country’s leading causes of death [1]. Non-communicable diseases (NCDs) such as cardiovascular disease (CVD), cancer, and chronic respiratory diseases have become top killers in the Chinese population, accounting for 86.6% of total deaths in 2013 [2]. In contrast, deaths from communicable diseases and injuries have decreased by 73.0% and 23.6% since 1990, respectively [2]. Unhealthful lifestyle factors, such as tobacco use, drinking, insufficient exercise, and imbalanced diet, are still prevalent in the population despite the behavior modification efforts made by public health practitioners to reduce these risk exposures in the past decades [3, 4]. Moreover, the prevalence of metabolic risk factors, including excess weight/obesity, hypertension, and hyperglycemia, has been increasing rapidly in the last decade [5–7]. If these risk factors are not well controlled, the disease burden of NCDs will continue to rise.

In 2011, the United Nations (UN) High-level Meeting on NCDs announced a worldwide initiative to prevent and control NCDs [8]. The World Health Organization (WHO) and its member states subsequently agreed on a target of a 25% reduction by 2025 in the probability of premature death among people aged 30–70 years for four main NCDs: CVD, diabetes, cancer, and chronic respiratory disease [9]. In September 2015, the UN General Assembly formally released the 2030 Agenda for Sustainable Development, which included a set of 17 bold new Global Goals and 169 specific targets. The agenda also includes the aim of reducing by one third premature mortality from NCDs compared to the current level [10]. To reach that goal, the WHO Global Monitoring Framework proposed ten specific targeted risk factors to be reduced [11]. The National Health and Family Planning Commission of the People’s Republic of China has stated strong support for these targets and is planning a corresponding national health policy, known as Health China 2030. Health China 2030 aims to adopt the most pertinent health policies or strategies for improving the overall health of the Chinese population over the next 15 years, and a core component is NCD prevention and control. In order to inform setting priorities when allocating limited resources in Health China 2030, this study has projected premature mortality from NCDs under different scenarios of risk factor reduction and assessed whether and how the goal of a one-third reduction in premature mortality from NCDs can be achieved.

## Methods

### Data

We used China results from the Global Burden of Disease Study (GBD) 2013 as empirical data for projection. Age-, sex-, and year-specific deaths between 1990 and 2013 for all NCDs and subcategories were extracted, along with population numbers. The standard methods of mortality estimation in GBD 2013 and special considerations for China are described in detail in our previous publications [2, 12]. In the present study, for ease of computation and interpretation, we projected premature mortality for CVD, cancer, chronic respiratory diseases, and diabetes that were aggregated from separate estimates for more specific subcategories and also combined into categories capturing minor or rare conditions. Additional file 1: Table S1 presented the categorization of NCDs in this study.

From the comparative risk assessment in GBD 2013, we used the estimates of risk factor exposure trends between 1990 and 2013 to project exposure level in 2030. GBD 2013 modeled age-sex-year exposure for 79 modifiable risk factors using all available data in China, including NCD and risk factor surveillance, nutrition surveys, health service surveys, epidemiological studies, and administrative data in China [13]. In the present study we included six risk factors which were included in the GBD study whose definitions harmonize with those of WHO, and we examined to what extent the mortality from NCDs and their main subcategories could decrease in 2030 if any or all risk factors meet the WHO targets. Those risk factors are systolic blood pressure (SBP, in millimeters of Hg), fasting plasma glucose (FPG, in millimoles/liter), body mass index (BMI, in kilograms/meter squared), total cholesterol (TC, in millimoles/liter), smoking (proportion of current smoking), and physical inactivity (defined as less than 600 metabolic equivalent minutes per week). The GBD 2013 adopted a set of unified relative risks (RRs) for all attribution analysis globally. The RRs were modeled using meta-regression with pool data from prospective cohort studies or published literature reviews [13], and those for the six risk factors and the subcause mortality of NCDs were extracted for the present study. All data were analyzed by sex, 5-year age group, and cause.

### Projection of NCD premature mortality in 2030

We projected NCD premature mortality by following three steps. The first step categorized all deaths into deaths that can be attributed to the aforementioned risk factors and unattributable parts, according to the theory of comparative risk assessment [13]. The second step projected risk factor exposure and unattributable deaths in 2030 assuming a constant annual change rate. We based this on the targets suggested by WHO on risk reduction to control NCDs [9]. Also in this step, we simulated eight separate scenarios to explore the potential effects of future risk factor reduction on premature mortality from NCDs. The reduction targets for those risk factors were taken directly from WHO’s voluntary global NCD targets [11], as the Chinese government has shown a strong interest in achieving them. The risk reduction was simulated differently for different risk factors. The prevalences of smoking and physical inactivity in 2030 were lowered to the WHO’s target levels in a straightforward way. For continuous exposure like cholesterol and SBP, risk reduction was made by shifting the population distribution to the left until the WHO’s target was met, while the distribution of BMI and fasting glucose was held constant to the 2013 level. Table 1 summarizes the specification of each scenario. Third, premature mortality for total NCDs and the main subcategories under each scenario were projected for 2030. Premature mortality was defined as the probability of dying between ages 30 and 70 years from NCDs and was estimated using age-specific death rates (in 5-year age groups between 30 and 70 years) with a life table method [14].The level in 2013 was considered as the baseline for the calculation of relative reduction in premature mortality. All data were prepared and analyzed in SAS 9.4. Additional file 1 documents details of the projection an﻿d t﻿he flowchart of the ﻿stu﻿dy design (Additional file 1: Figure S1).

**Table 1:** Scenario specifications in risk factor exposure projection according to the WHO Global Monitoring Framework

Scenario	Scenario specification
Natural trend	Age and sex-specific risk factor exposures were projected assuming the annual change rate remained similar to that between 1990 and 2013.
Smoking	Age and sex-specific prevalences of smoking in 2030 are reduced relatively by 30% from the 2013 level. All other risk factors follow the natural trends.
Physical inactivity	Age and sex-specific prevalences of physical inactivity in 2030 are 10% relatively less than in 2013. All other risk factors follow the natural trends.
High BMI	Age and sex-specific distributions of BMI in 2030 are the same as in 2013. All other risk factors follow the natural trends.
Total cholesterol	Age and sex-specific distributions of total cholesterol are shifted in 2030 so that the prevalence of raised cholesterol (defined as ≥5.0 mmol/L) is reduced relatively by 20%. All other risk factors follow the natural trends.
Fasting glucose	Age and sex-specific distributions of fasting glucose in 2030 are the same as in 2013. All other risk factors follow the natural trends.
Systolic blood pressure (SBP)	Age and sex-specific distributions of SBP are shifted in 2030 so that the prevalence of raised SBP (defined as ≥140 mm Hg) is reduced relatively by 25% from the 2013 level﻿. All other risk factors follow the natural trends.
All targets are achieved in 2030	All targets described above are achieved in 2030.

### Presentation of the analysis results

We firstly presented the death numbers, mortality rate, and probability of dying between ages 30 and 70 years by NCD causes in 2013, and those statistics in 2030, provided that all study risk factors continued their past trends. We also examined the relative changes in between to show to what extent those indicators would increase or decrease in the future without reinforced intervention on risk reduction (Table 2). Next, we estimated the absolute number of premature deaths that would be avoided if any or all risk reduction targets were achieved, so as to compare the absolute health benefit that would be harvested among different risk factor strategies. Finally, trends of premature mortality by main NCD causes between 2013 and 2030 in the eight risk reduction scenarios were shown to visually assess the feasibility of a one-third reduction in premature mortality due to NCDs in China.

**Table 2:** Deaths and premature mortality of main NCDs for people aged 30–70 in 2013 and projections for 2030 if risk factor trends continue in China

Gender	Disease	2013			2030			Percent change		
		Deaths (in thousands)	Mortality rate^a^ (1/100,000)	Premature mortality^b^ (%)	Deaths (in thousands)	Mortality rate if risk factor trends continue (1/100,000)	Premature mortality if risk factor trends continue (%)	Deaths	Mortality rate	Premature mortality
Both	Total	3108	432.2	19.8	3521	431.8	17.2	13.3	–0.1	–13.1
	CVD	1241	172.6	8.6	1518	186.2	7.8	22.3	7.9	–9.3
	Cancer	1270	176.6	8.3	1444	177.1	7.5	13.7	0.3	–9.6
	Diabetes mellitus	59	8.1	0.4	75	9.2	0.4	27.1	13.6	0
	Chronic respiratory diseases	210	29.3	1.7	165	20.2	0.9	–21.4	–31.1	–47.1
	Other NCDs	328	45.6	2.1	319	39.1	1.7	–2.7	–14.3	–19
Men	Total	2043	553.6	25.4	2453	586	23.5	20.1	5.9	–7.5
	CVD	806	218.5	11.2	1041	248.6	10.8	29.2	13.8	–3.6
	Cancer	847	229.6	11.2	1026	245.1	10.6	21.1	6.8	–5.4
	Diabetes mellitus	30	8.3	0.4	43	10.2	0.5	43.3	22.9	25
	Chronic respiratory diseases	138	37.5	2.3	119	28.5	1.3	–13.8	–24.0	–43.5
	Other NCDs	222	59.8	2.8	224	53.6	2.4	0.9	–10.4	–14.3
Women	Total	1065	304.2	13.4	1068	269.1	10.2	0.3	–11.5	–23.9
	CVD	435	124.3	5.9	478	120.4	4.6	9.9	–3.1	–22
	Cancer	422	120.7	5.3	418	105.3	4.3	–0.9	–12.8	–18.9
	Diabetes mellitus	28	8	0.4	32	8.2	0.3	14.3	2.5	–25
	Chronic respiratory diseases	72	20.6	1.1	45	11.4	0.4	–37.5	–44.7	–63.6
	Other NCDs	108	30.6	1.4	95	23.9	1	–12	–21.9	–28.6

^a^Mortality rate was calculated by dividing deaths by population number.

^b^Premature mortality is the probability of dying between ages 30 and 70 years from specific cause that was calculated using life table method.

## Results

### Premature deaths and mortality in 2030

If past trends for each risk factor continue to 2030, the total annual premature deaths due to NCDs would increase from 3.11 million to 3.52 million, with a decrease in the premature mortality rate of 13.1% (Table 2). The premature deaths from CVD would increase from 1.24 million in 2013 to 1.52 million in 2030, accounting for the largest portion (277,000 deaths) of the total increase in NCD premature deaths. This would be followed by cancer (174,000) and diabetes (16,000), while premature deaths from chronic respiratory diseases would decrease by 45,000. The risk of a premature death in 2030 would be highest for CVD (7.8%) and cancer (7.5%).

The mortality rate of all NCDs remains similar between 2013 (432.2 per 100,000 people) and 2030 (431.8/100,000). With regard to specific causes, the mortality rate would increase fastest in diabetes (by 13.6% relatively) and CVD (7.9%), and chronic respiratory diseases and other NCDs would see a relative decrease of 31.1% and 14.3%, respectively, while cancer in 2030 would still remain at the 2013 level.

The premature mortality for each NCD subcategory was consistently higher in men than in women, with differences of more than twofold. The percentage of premature mortality from chronic respiratory disease would undergo a 47.1% relative decrease between 2013 and 2030, and the probability for CVD and cancer would observe 9.3% and 9.6% relative decreases, respectively, while the rate for diabetes would be unchanged. Most NCD subcategories saw a decrease in premature mortality, but the magnitude varied wildly. For women, premature mortality from NCDs decreased, with the sharpest being a 63.6% decline in deaths from chronic respiratory diseases. Decreases among women in premature mortality from CVD (22.0%), cancer (18.9%), and diabetes (25.0%) were also notable. In contrast, only small decreases were seen in male premature mortality from CVD (3.6%) and cancer (5.4%), although a 43.5% decline was predicted in chronic respiratory diseases. Additional file 1: Table S1 further presents the estimates of deaths and premature mortality for each specific NCD subcategory.

### Premature deaths avoided in multiple scenarios

In the combined scenario in which all risk factor reduction targets are achieved, nearly one million deaths due to NCDs among 30- to 70-year-olds would be avoided compared with the projection should current trends continue (Table 3). Most of the avoided deaths are due to CVD (700,000), followed by cancer (240,000), chronic respiratory disease (36,000), and diabetes (15,000). Many more deaths are avoided in men (730,000) in the combined scenario than in women (260,000).

**Table 3:** Reduction in deaths for four main NCDs in 2030 with different risk factor scenarios (in thousands)

		Smoking	Physical activity	High BMI	Fasting glucose	Total cholesterol	SBP	All targets achieved
Both	Total	326.0	7.8	95.6	57.4	52.7	564.1	998.8
	CVD	66.6	6.7	54.9	57.4	52.7	564.1	703.1
	Cancer	222.4	0.6	26.9	0	0	0	244.8
	Diabetes mellitus	1.2	0.4	13.8	0	0	0	15.1
	Chronic respiratory diseases	35.8	0	0	0	0	0	35.8
Men	Total	280.1	7.1	67.4	39.2	37.3	391.9	734.8
	CVD	75.0	6.2	37.1	39.2	37.3	391.9	503.3
	Cancer	178.7	0.5	22.8	0	0	0	197.6
	Diabetes mellitus	1.2	0.4	7.5	0	0	0	8.8
	Chronic respiratory diseases	25.2	0	0	0	0	0	25.2
Women	Total	45.9	0.7	28.2	18.1	15.4	172.2	264.0
	CVD	–8.4	0.6	17.8	18.1	15.4	172.2	199.8
	Cancer	43.8	0.1	4.1	0	0	0	47.3
	Diabetes mellitus	0	0.1	6.3	0	0	0	6.3
	Chronic respiratory diseases	10.6	0	0	0	0	0	10.6

As a single target, reduction in the prevalence of hypertension provided the largest reduction in premature deaths for both genders (390,000 in men and 170,000 in women), all in CVD. A reduction in smoking prevalence had the second largest impact, particularly on lung cancer deaths among males. However, reducing smoking prevalence by only 30% would result in more CVD deaths in women, as the projected current trend in smoking prevalence leads to a decrease of more than 30% by 2030. Compared with the current trend, a halt in the rise of average BMI could prevent 95,600 extra deaths, mainly in CVD (54,900) and cancer (26,900). There were also notable benefits of achieving the targets for fasting glucose and total cholesterol, with 57,400 and 52,700 deaths avoided, respectively. A modest impact on deaths was seen when the prevalence of physical inactivity decreased by 10%. In all simulated scenarios, more deaths in men were saved than in women for all NCD categories (Table 3). Estimates of premature deaths in NCD subcategories are shown in Additional file 1: Table S2.

### Achievability of the one-third reduction goal in premature mortality from NCDs

**Fig. 1:**
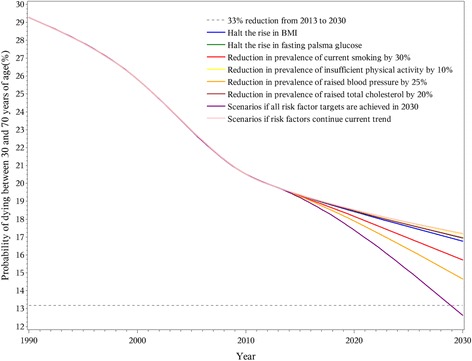
Probability of premature death due to NCDs between ages 30 and 70 in China from 1990 to 2030

If all risk factor targets are achieved, the premature mortality from NCDs for people aged 30–70 could be reduced by more than 33% between 2013 and 2030 (Fig. 1). However, reduction in any one risk factor could not meet the goal of a one-third reduction in premature mortality.

**Fig. 2:**
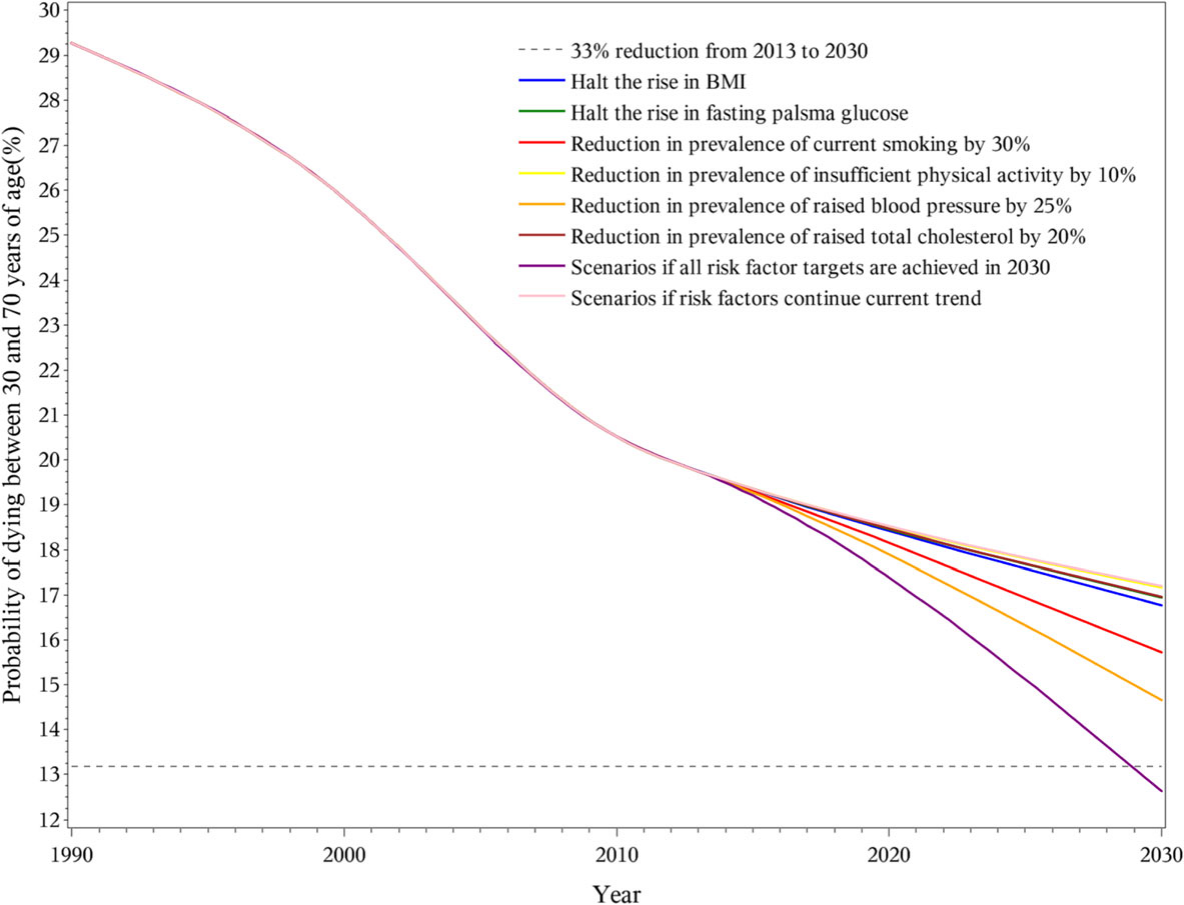
Probability of premature death due to cardiovascular disease between ages 30 and 70 in China from 1990 to 2030

**Fig. 3:**
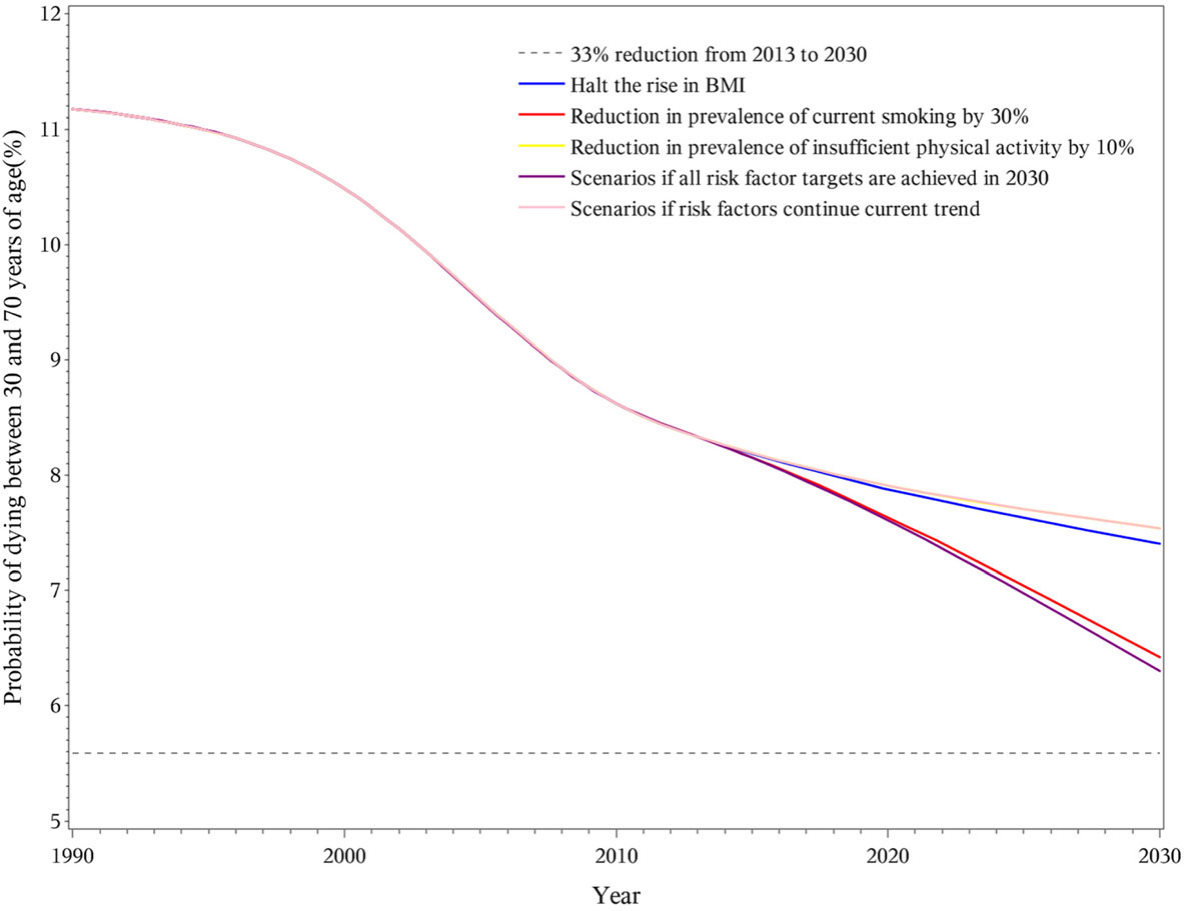
Probability of premature death due to cancer between ages 30 and 70 in China from 1990 to 2030

**Fig. 4:**
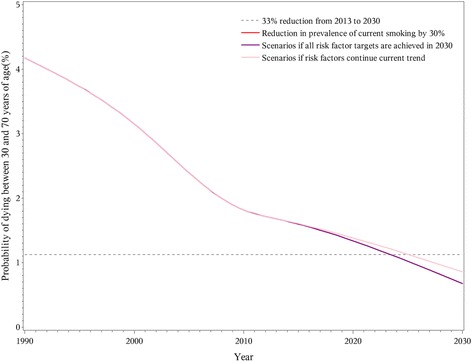
Probability of premature death due to chronic respiratory disease between ages 30 and 70 in China from 1990 to 2030

**Fig. 5:**
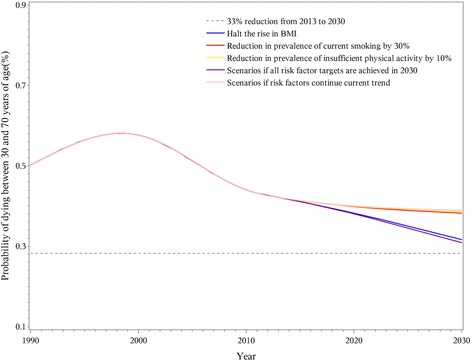
Probability of premature death due to diabetes mellitus disease between ages 30 and 70 in China from 1990 to 2030

Specifically, the 33% premature mortality reduction in 2030 was achieved for CVD and chronic respiratory diseases, but not for cancer and diabetes, even in the combined scenario where all risk factor targets were reached. A 25% reduction in the prevalence of hypertension had the largest impact on CVD premature mortality and could achieve the goal even if no other risk factors were changed (Fig. 2). Other CVD-related risk factors provided relatively small contributions to reaching the goal. For cancer, the goal of a 33% reduction in premature mortality was not achieved even if all targets for related risk factors were reached by 2030 (Fig. 3). With the continuation of current trends, reduction in premature mortality from chronic respiratory disease in 2030 would surpass the goal (Fig. 4). The decline would be greater with a 30% reduction in overall smoking prevalence for both genders. While the 33% reduction goal would not be accomplished for diabetes even if all risk factors were achieved, the decrease in premature mortality from this cause would still be notable. A halt in the rise of average BMI was a particularly large component of the decrease in diabetes (Fig. 5).

Scenario projections by sex were also performed and are shown in Additional file 1: Figures S2–S11. Given the higher baseline premature mortality in men than in women, risk factor reduction in men achieved sharper declines in premature mortality for all NCDs than it did among women. A gender difference was also found in the reduction of diabetes premature mortality: women saw a target-achieving decline if all risk factor targets were met, while men did not. Trend of risk factor exposure by age and sex between 1990 and 2030 are documented in Additional file 1: Tables S5-S10.

## Discussion

Ours is the first study to attempt to provide answers to questions raised during the design of the Health China 2030 program on future trends in premature mortality from NCDs. The findings suggest that a large reduction in these deaths is possible by 2030 if certain targets for risk factor intervention are reached. The UN goal of an overall one-third reduction in premature mortality due to NCDs could be successfully achieved by meeting the organization’s targets for related risk factors, but meeting the goal for cancer and diabetes would require extra efforts. These results provide rich information for setting priorities when allocating limited resources in the Health China 2030 program.

The present study suggests that, despite the fact that mortality from most NCDs is already declining and a great increase in life expectancy has been achieved in China over the past two decades [2], one million premature deaths due to NCDs could still be avoided in 2030 if the exposure levels of a small number of risk factors were reduced. Loss of productivity due to aging and premature deaths from NCDs is considered a major barrier to poverty alleviation and sustainable development [14]. Simulations in this study showed it is possible to reach the UN goals for reducing premature mortality from NCDs by the year 2030. The prerequisite for this success is to reduce the population’s exposure to high-impact risk factors, particularly high SBP, smoking, and high BMI.

Although adequate reduction in high SBP would have a notable impact on premature mortality, there remain a number of important obstacles to population-based blood pressure management and control. Recent national surveys show a consistent and rapid increase in hypertension prevalence, from 18.0% in 2002 to 27.8% in 2013 [15, 16]. Despite joint efforts by public health practitioners and medical professionals in the last decade, awareness, treatment, and control of hypertension remain poor. In 2013, 40.9% of hypertensive individuals were aware of their condition, 32.5% were receiving antihypertensive medication, and only 9.7% had their blood pressure controlled (defined as SBP ≤140 mm Hg and diastolic blood pressure ≤90 mm Hg) [16]. To reach the WHO’s target of reducing hypertension prevalence by 25%, efforts should be concentrated on two important aspects: prevention and management. Lifestyle modification is the most important strategy in preventing hypertension. However, hypertension-related risk factors remained at a prevalent level in the last decade, including smoking, a diet high in calories from fat, physical inactivity, and excess weight or obesity [16]. To keep the blood pressure of a hypertensive population within the normal range requires early detection of the condition, good compliance with appropriate antihypertensive treatment, regular visits to doctors, necessary lifestyle modifications, and a robust primary health system [17–19]. However, the situation in China is not promising, particularly in the vast rural areas and underdeveloped urban cities. Therefore, achieving an overall 25% reduction in the prevalence of high blood pressure in the next 15 years presents a significant challenge for the public health system.

Smoking is also a serious and complicated public health problem. The global Adult Tobacco Survey in 2010 reported that 53% of Chinese men aged 15 years and older were current smokers, though the rate was much lower for women (2.4%) [20]. The smoking prevalence among men has decreased only slightly since 1996 [21]. Intervention to promote smoking cessation seems ineffective, as smokers quit smoking mainly because of poor health [21, 22]. In addition, although smoking cessation clinics have been rapidly expanded in urban hospitals, few smokers visit them and seek help [23]. China officially ratified the WHO’s Framework Convention on Tobacco Control (FCTC) in 2006, but progress has been slow due to multiple political, social, and economic obstacles [24]. The implementation of “the tax linkage,” which increased the cigarette wholesale and valorem tax rate from 5% to 11%, was not implemented until 2015, nearly ten years after the ratification of the FCTC [25]. However, the increase in tax still seems very modest compared to that in developed countries; e.g., the price of cigarettes in France grew by 44.7% in 2003–2004 [26]. China is still one of the countries where the price of cigarettes is very cheap [25], although raising taxation has been proven as an effective measure to reduce tobacco consumption [27]. The use of large pictorial health warnings on cigarette packages is a highly cost-effective way to inform the public about the health risks of tobacco consumption, but its implementation in China is still far from satisfactory [23]. Until early 2017, only 18 cities had legislated to ban smoking in public areas, and the public law enforcement is rare to non-existent outside of the most highly developed cities like Beijing, Shanghai, and Shenzhen. More efforts are still needed to overcome various barriers to reaching the FCTC goals.

The prevalence of excess weight or obesity has seen steady growth in China for nearly 20 years [28, 29], mainly because of a transformation in dietary patterns and a reduction in physical activity driven by the growth of modern transport [30]. The Chinese government is fighting an epidemic of obesity in the younger generation. A nationwide campaign, “Eat smart at school,” aims to promote and cultivate a long-term healthful lifestyle in the educational setting. There are also a number of nationwide health promotion campaigns that have incorporated new techniques, such as Internet-based wearable devices or smartphone apps which drive physical activity among users. Despite these public health interventions, the target of halting the rise in overweight and obese people between 2013 and 2030 might only be reached with sustained health education and promotion in the population with appropriate techniques in this country where an obesity epidemic prevails.

We validated the current projection with other methods or assumptions. In the scenario where current trends continue, premature deaths from NCDs increased from 3.2 million to 3.5 million. With the expected increased burden of NCDs over the next 15 years, an increase of only 300,000 premature deaths in such a populous country may be an underestimate. We, therefore, additionally projected deaths for all ages with the same methods adopted in the present study. The total number of deaths in 2013 for NCDs was 8.5 million, which would increase by 43% to 12.2 million in 2030 (Additional file 1: Table S3). It is clear that the majority of deaths from NCDs would occur in people older than 70. An aging population, increasing life expectancy, and improving medical techniques might be important reasons for this. To validate the 2030 projection of NCDs deaths in the scenario with no interventions, we further produced estimates assuming NCD mortality trends continued to 2030 with a constant change rate (proportional change model). We saw very similar results (Additional file 1: Table S4). There was a difference of only 190,000 (5% relative difference) in this projection of total NCD deaths and a 0.8% difference in overall premature mortality. The projection in the present study, we believe, is reliable.

Our study suffered from several limitations. First, the projection relied heavily on estimates driven by GBD 2013; therefore, all the limitations in estimates of deaths, mortality, and attributable burden in the GBD study apply to this analysis. We noticed that RRs for the Chinese population might well differ from global RRs adopted by GBD 2013, and thus the attributable burden of risk factors for China might be biased. However, China-specific estimations of associations between study risk factors and related NCD mortality are still comparatively rare and embryonic, and it is difficult to anticipate the direction and magnitude of these associations until more solid evidence appears. As more and more large population-based cohort studies are emerging in China, e.g., China Kadoorie Biobank [31], the Shanghai Men's and Women's Health Study Cohorts [32, 33], and the Taizhou Longitudinal Study [34], more compelling evidence on China-specific RRs would be available in the future and could be potentially adopted in the future GBD study for China’s specific estimation. Second, as diabetes is 100% attributed to high fasting glucose, and hypertensive heart disease is 100% attributed to high SBP in the comparative risk assessment framework of GBD 2013, there were no unattributable deaths for these risk factors in the present study. Theoretically, the present projected deaths and premature mortality in diabetes and CVD must have been underestimated in the scenarios of fasting glucose, systolic blood pressure, and the combined scenario. However, as the absolute number of deaths for diabetes and hypertensive heart disease is quite small relative to total deaths from all NCDs, this should have little impact on the conclusion. Third, some important risk factors for NCDs were not included in the present study, such as sodium intake, other dietary risk factors, and environmental risk factors. We excluded sodium because the causal relationship between salt intake and SBP makes SBP a perfect substitute in the attribution analysis. Even if we included sodium, its impact on CVD would be erased, as SBP completely mediates that impact. WHO did not set goals for reducing cancer-related dietary and environmental risk factors, so we did not simulate scenarios for these risk factors. This may partly explain why premature mortality from cancer failed to reach the 33% reduction goal in the risk factor reduction scenarios. Fourth, duration of exposure is usually required before outcome occurs, and the time lag in between varies for different exposure-outcome pairs. For example, smoking rapidly increases RR for CVD but only does so slowly for lung cancer or chronic obstructive pulmonary disease, and the same pattern is also observed among those who quit. This was extensively discussed in the GBD study on risk factor burden [13]. As the differences are already captured in the mortality trends for China estimated for the GBD in the present study, we implicitly assume that the same time durations for exposure and disease outcomes will continue into the future.

## Conclusions

The absolute burden of premature deaths due to NCDs will continue to increase over the next decade and a half. Effective modification of NCD-related risk factors to meet WHO’s targets could not only prevent the burden from increasing but also help China achieve the UN Sustainable Development Goal of a one-third reduction in premature mortality from NCDs. However, reinforcement of public health interventions is needed to secure the risk factor reduction.

## Additional file

Additional file 1: Online material providing details of projection of NCD mortality, flowchart of the study design, and data processing (**Figure S1**), figures of projections by sex (**Figures S2–S11**), and supplemental tables (**Tables S1**–**S10**). (PDF 1081 kb)

## Abbreviations

BMI: Body mass index
CVD: Cardiovascular disease
FPG: Fasting plasma glucose
GBD: Global Burden of Disease study
NCD: Non-communicable disease
RR: Relative risk
SBP: Systolic blood pressure
TC: Total cholesterol
UN: United Nations
WHO: World Health Organization
