# Supplementary material for: Can China achieve a one-third reduction in premature mortality from non-communicable diseases by 2030?
Source: BMC Med. 2017 Jul 11;15:132. doi: 10.1186/s12916-017-0894-5 (PMC5504650; doi:10.1186/s12916-017-0894-5)
Supplement: Additional file 1: — Online material providing details of projection of NCD mortality, flowchart of the study design, and data processing (Figure S1), figures of projections by sex (Figures S2–S11), and supplemental tables (Tables S1–S10). (PDF 1081 kb) [file 12916_2017_894_MOESM1_ESM.pdf]

Appendix to Can China Achieve a One-third Reduction in Premature Mortality from Non-communicable Diseases by 2030?

This appendix provide more methodological detail, plots of projection by sex, and results of validation analysis.

Table of Contents

Projection of NCD mortality in 2030 2
Additional file Figures 5
Additional file Tables 16

Projection of NCD mortality in 2030

Projection of NCD mortality was conducted in three steps for each age-sex-cause-year group. First, we categorized all deaths into deaths that can be attributed to the aforementioned risk factors and non-attributable parts, according to the theory of comparative risk assessment.[1] For continuous risk factors, such as BMI, SBP, TC, and FPG, the formula for the population attributable fraction (PAF) is as follows:

$$\text{PAF} = \frac{\int_{x=0}^{m} RR(x)P1(x)\,dx - \int_{x=0}^{m} RR(x)P2(x)\,dx}{\int_{x=0}^{m} RR(x)P1(x)\,dx}$$

where $RR(x)$ is the RR of a certain disease for exposure level *x*, $P1(x)$ is the population distribution of the exposure, $P2(x)$ is the minimum theoretical exposure distribution, and m is the maximum exposure level. The minimum theoretical exposure is the counterfactual condition of exposure, and was defined previously for each risk factor.[2]

With regard to categorical risk factors (smoking and physical inactivity), the formula for PAF is:

$$\text{PAF} = \frac{\sum_{i=1}^{n} P_i(RR_i - 1)}{\sum_{i=1}^{n} P_i(RR_i - 1) + 1}$$

Where *i* is exposure level, RR_i is RR for exposure level *i*, and P_i is the prevalence of exposure level *i*.

Estimated PAFs divided total deaths into attributable and non-attributable parts. The attributable part was calculated by multiplying PAF and total deaths, while the non-attributable part was total deaths minus the attributable part. Deaths attributable and non-attributable to NCDs were estimated by age, sex, and year separately between 1990 and 2013.

The second step was to project risk factor exposure and non-attributable deaths in 2030. A proportional change model was adopted to estimate the annual change rate in risk factor exposure and non-attributable deaths from 1990 to 2013. Assuming the observed trends continue similarly into the future, we obtained estimates of exposure in 2030 using the following formula with constant annual change rate:

$$Exposure_{2030} = exposure_{2013} * \exp(\frac{\ln\left(\frac{exposure_{2013}}{exposure_{1990}}\right)}{23} * 17)$$

With projected exposure in 2030 and RRs from GBD 2013, we estimated PAF in 2030 for each risk-outcome pair. Non-attributable deaths for a specific disease in 2030 was projected similarly by replacing exposure level with non-attributable deaths in the proportional change model. The total deaths in 2030 for the disease was then projected by the following formula:

$$Total\ deaths_{2030} = (unattributable\ deaths_{2030}/(1 - PAF_{2030}))$$

Based on the targets suggested by WHO on risk reduction to control NCDs,[3] we simulated eight separate scenarios to explore the potential effects of risk factor reduction on premature mortality from NCDs. Table 1 summarizes each scenario. For the scenario in which all targets are achieved, we

calculated the joint PAF of all risk factors using the following formula. The mediation factors were estimated using GBD 2013, [1] as some behavioral risk factors are mediated through other factors, e.g., a certain degree of hazard in ischemic heart disease associated with obesity is mediated through elevated fasting plasma glucose and total cholesterol.

$$PAF_{oast} = 1 - \prod_{j=1}^{J} (1 - PAF_{ioast} \prod_{i=1}^{J} (1 - MF_{jio}))$$

Where J is the number of risk factors for calculating the joint effect, PAF_{ioast} is the attributable fraction of i risk factor, MF_{jio} is the mediation factor between risk factor i and a certain disease o through risk factor j, a was the age group, s was sex, and t was year.

Third, premature mortality for total NCDs and the main subcategories under each scenario were projected for 2030. Premature mortality was defined as the probability of dying between ages 30 and 70 years from NCDs and was estimated using age-specific death rates (in 5-year age groups between 30 and 70 years) with a life table techniques with the following formulas. [4]

First, five-year death rates (${}_{5}^{*}\mathrm{M}_{\mathrm{x}}$) was calculated:

$${}_{5}^{*}\mathrm{M}_{\mathrm{x}} = \frac{\text{Total deaths from a specific disease between exact age } x \text{ and exact age } x + 5}{\text{Total population between exact age } x \text{ and exact age } x + 5}$$

For each five-year age group, the probability of death from the disease (${}_{5}^{*}q_{\mathrm{x}}$) was calculated：

$${}_{5}^{*}q_{\mathrm{x}} = \frac{{}_{5}^{*}\mathrm{M}_{\mathrm{x}} * 5}{1 + {}_{5}^{*}\mathrm{M}_{\mathrm{x}} * 2.5}$$

The unconditional probability of death, for the 30-70 age range, was calculated last：

$${}_{40}^{*}q_{30} = 1 - \prod_{\mathrm{x}=30}^{65} (1 - {}_{5}^{*}q_{\mathrm{x}})$$

The age-specific death rate under each scenario was calculated as the estimated deaths divided by the population. The sex- and age-specific population in 2030 was projected by the Population Division of United Nations (UN).

All data were prepared and analyzed in SAS 9.4. Efigure 1 shows the flowchart of this study.

References

1. Forouzanfar MH, Alexander L, Anderson HR, et al. Global, regional, and national comparative risk assessment of 79 behavioural, environmental and occupational, and metabolic risks or clusters of risks in 188 countries, 1990-2013: a systematic analysis for the Global Burden of Disease Study 2013. *Lancet* 2015; **386**(10010): 2287-323.
2. Lim SS, Vos T, Flaxman AD, et al. A comparative risk assessment of burden of disease and injury attributable to 67 risk factors and risk factor clusters in 21 regions, 1990-2010: a systematic analysis for the Global Burden of Disease Study 2010. *Lancet* 2012; **380**(9859): 2224-60.
3. WHO. Monitoring framework and targets for the prevention and control of NCDs. Revised WHO discussion paper on the development of a comprehensive global monitoring framework, including indicators, and a set of voluntary global targets for the prevention and control of NCDs. July 25, 2012. http://www.who.int/nmh/events/2012/ncd_discussion_paper/en (accessed April 10, 2016) .
4. World Health Organization. Global status report on noncommunicable diseases 2014. 2014. Geneva. WHO Press.

Additional file Figures

Figure S1 Flowchart of the study design and data processing*.

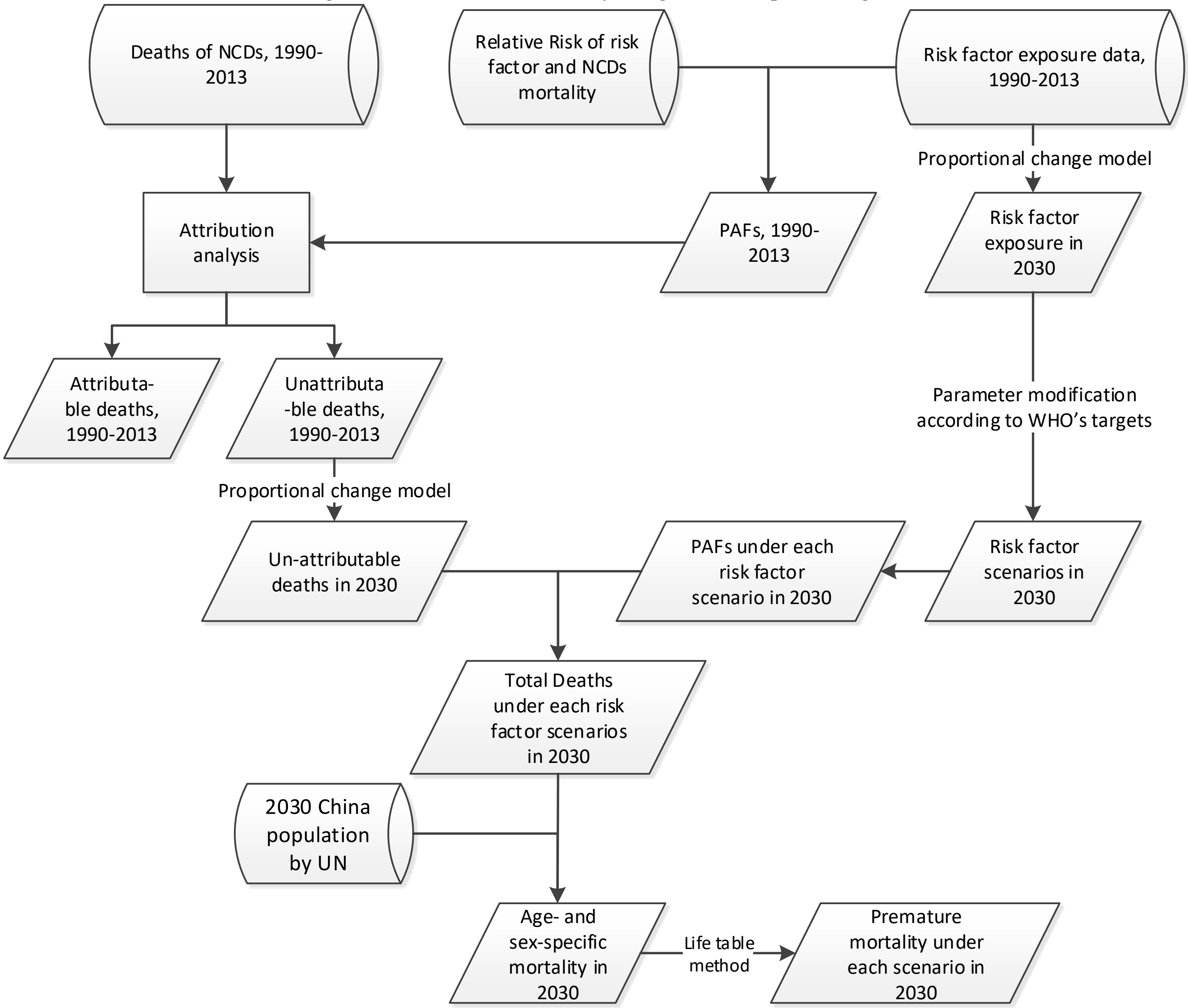

*All analyses were performed separately by age, sex, year, and NCD causes.

Figure S2

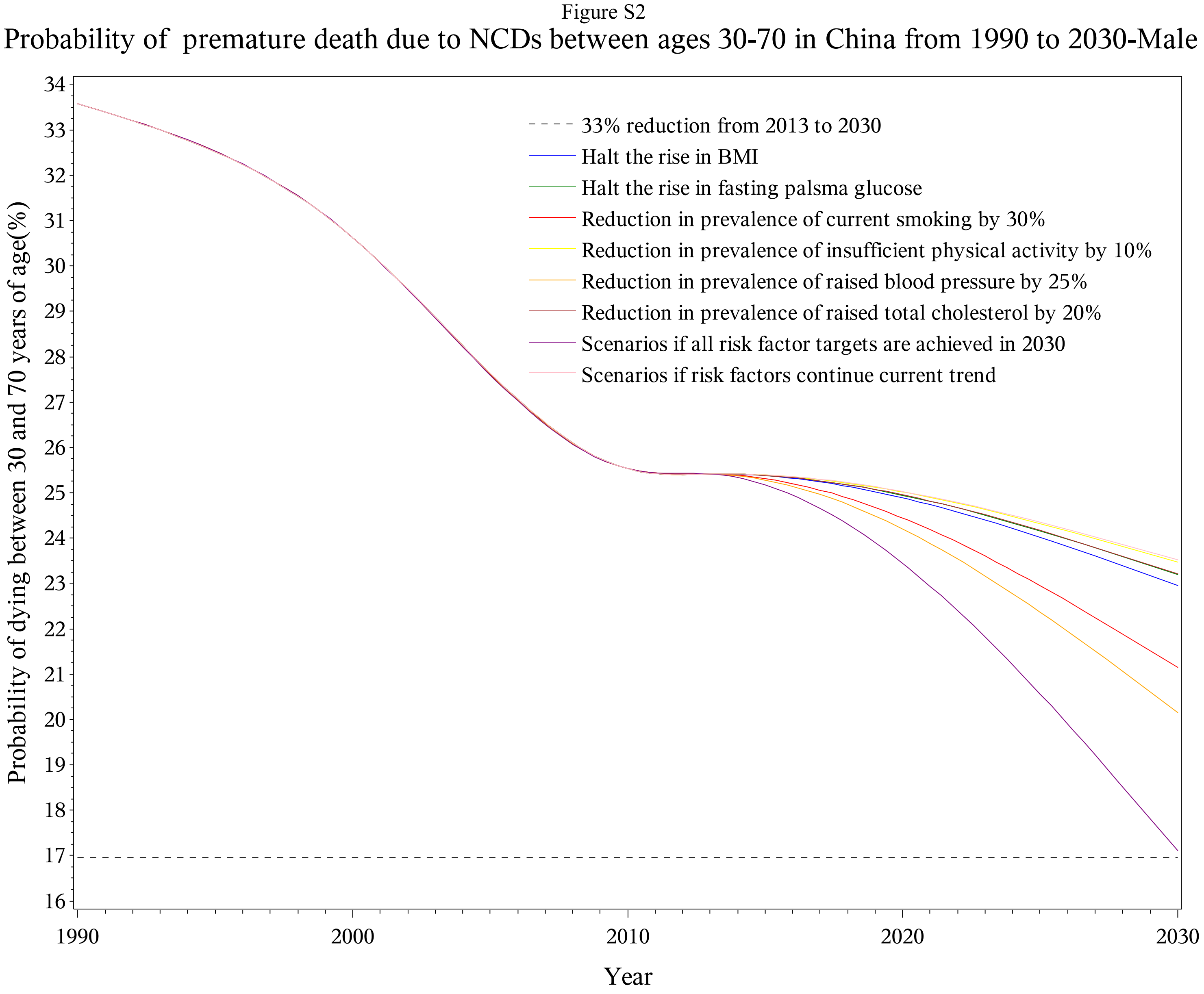

Figure S3

Probability of premature death due to NCDs between ages 30-70 in China from 1990 to 2030-Female

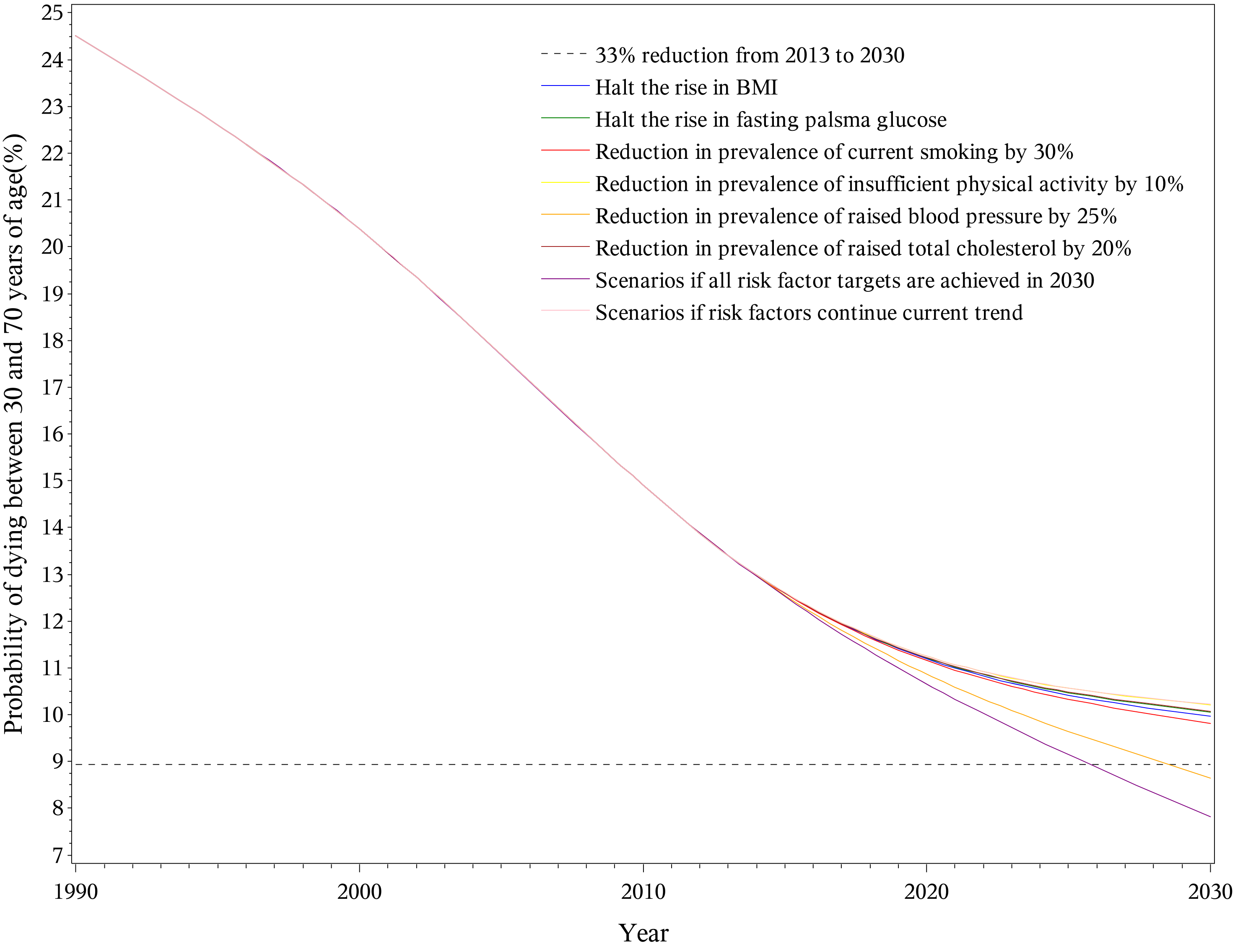

Figure S4

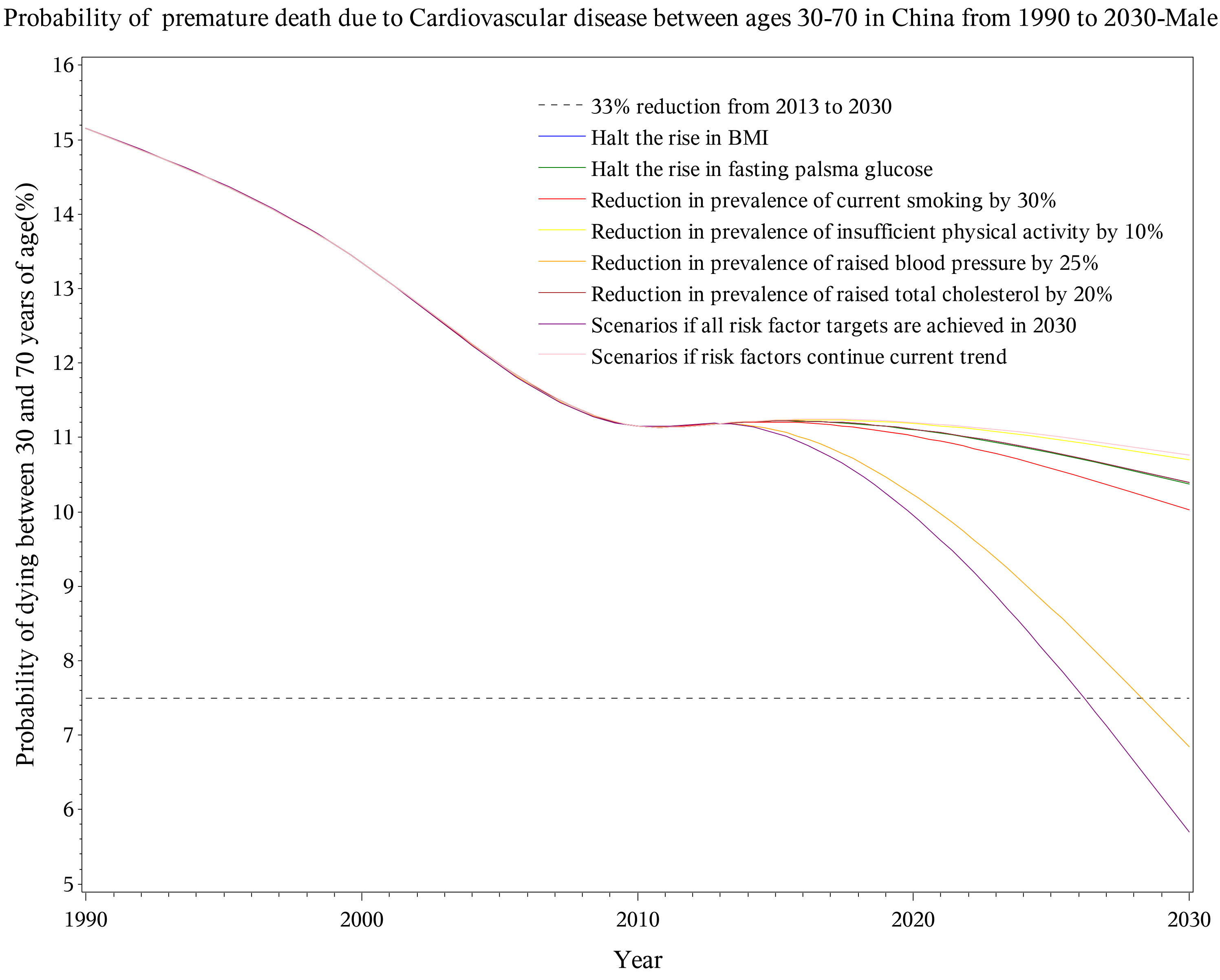

Probability of premature death due to Cardiovascular disease between ages 30-70 in China from 1990 to 2030-Male
33% reduction from 2013 to 2030
Halt the rise in BMI
Halt the rise in fasting palsma glucose
Reduction in prevalence of current smoking by 30%
Reduction in prevalence of insufficient physical activity by 10%
Reduction in prevalence of raised blood pressure by 25%
Reduction in prevalence of raised total cholesterol by 20%
Scenarios if all risk factor targets are achieved in 2030
Scenarios if risk factors continue current trend
Probability of dying between 30 and 70 years of age(%)
16
15
14
13
12
11
10
9
8
7
6
5
1990
2000
2010
2020
2030
Year

Figure S5

Probability of premature death due to Cardiovascular disease between ages 30-70 in China from 1990 to 2030-Female

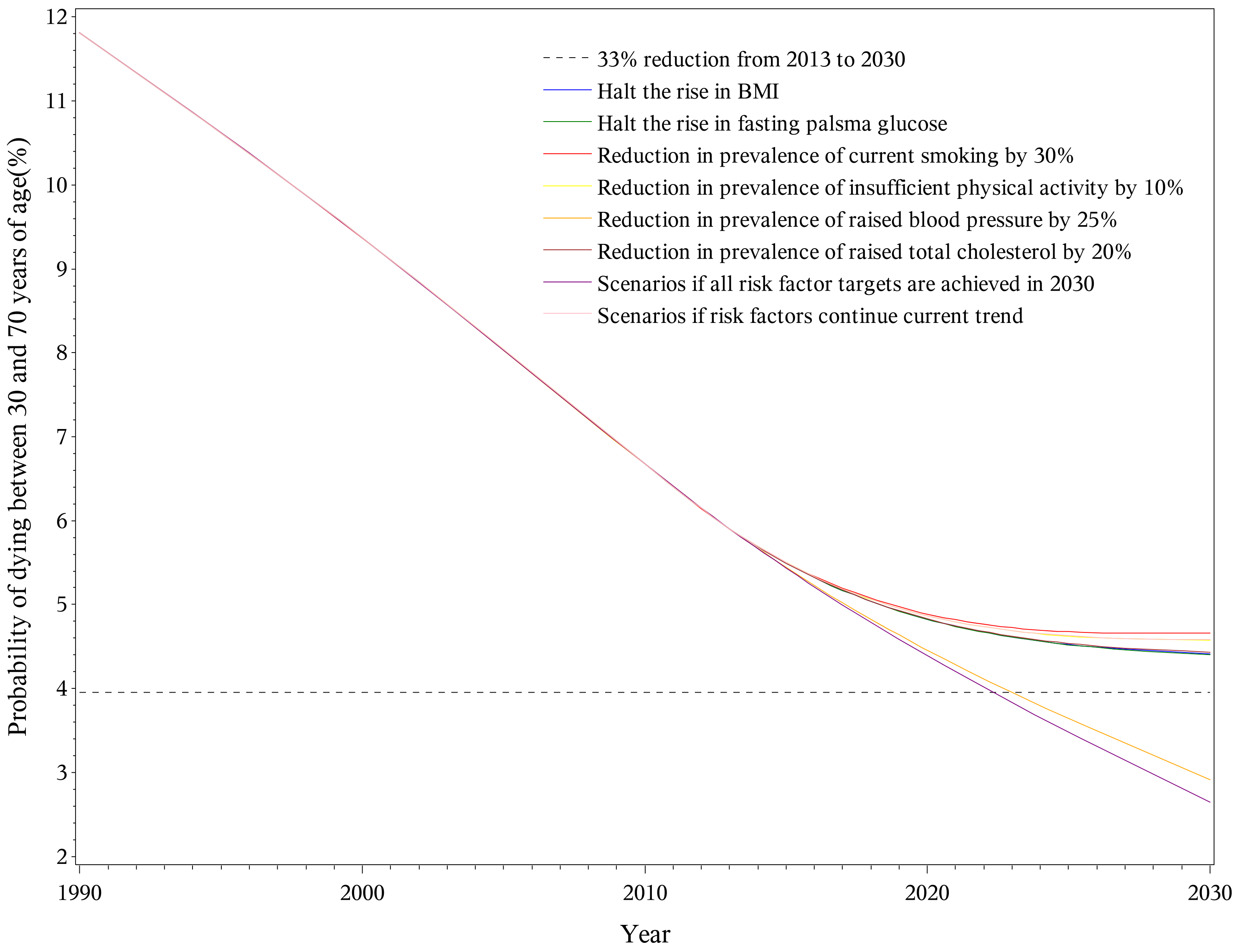

Figure S6

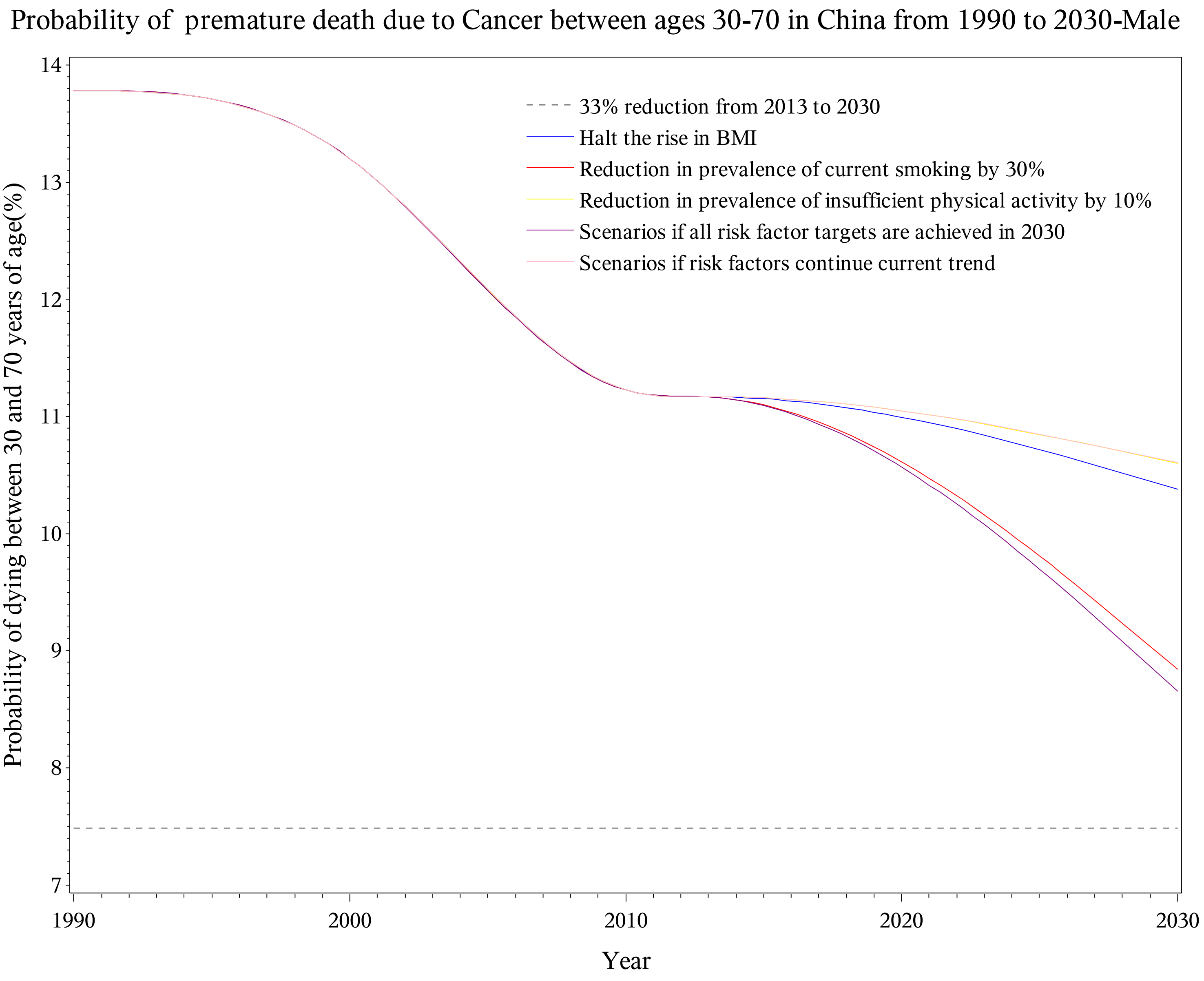

Probability of premature death due to Cancer between ages 30-70 in China from 1990 to 2030-Male
33% reduction from 2013 to 2030
Halt the rise in BMI
Reduction in prevalence of current smoking by 30%
Reduction in prevalence of insufficient physical activity by 10%
Scenarios if all risk factor targets are achieved in 2030
Scenarios if risk factors continue current trend
Probability of dying between 30 and 70 years of age(%)
14
13
12
11
10
9
8
7
1990
2000
2010
2020
2030
Year

Figure S7

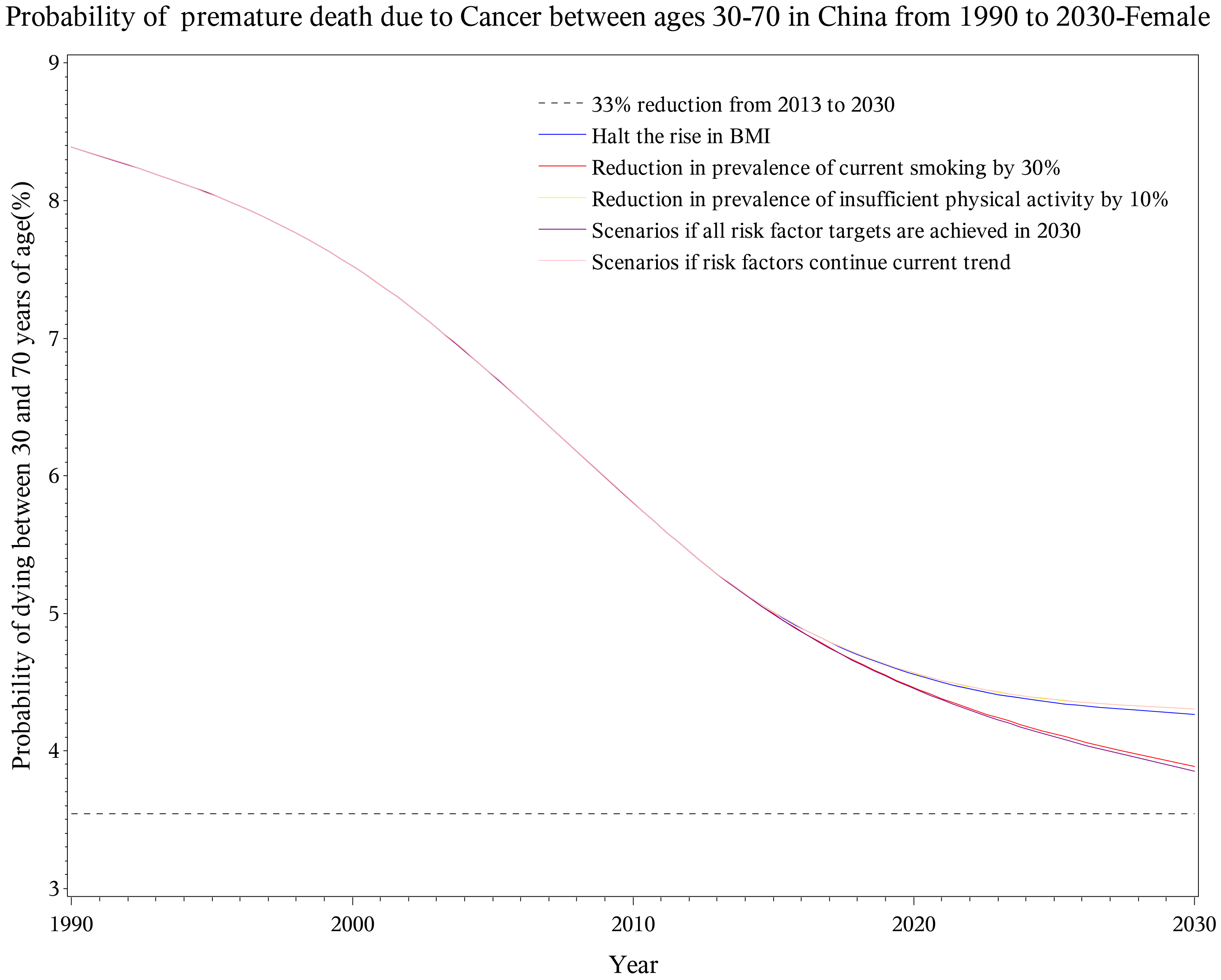
Probability of premature death due to Cancer between ages 30-70 in China from 1990 to 2030-Female
33% reduction from 2013 to 2030
Halt the rise in BMI
Reduction in prevalence of current smoking by 30%
Reduction in prevalence of insufficient physical activity by 10%
Scenarios if all risk factor targets are achieved in 2030
Scenarios if risk factors continue current trend
Probability of dying between 30 and 70 years of age(%)
9
8
7
6
5
4
3
1990
2000
2010
2020
2030
Year

Figure S8

Probability of premature death due to Chronic respiratory disease between ages 30-70 in China from 1990 to 2030-Male

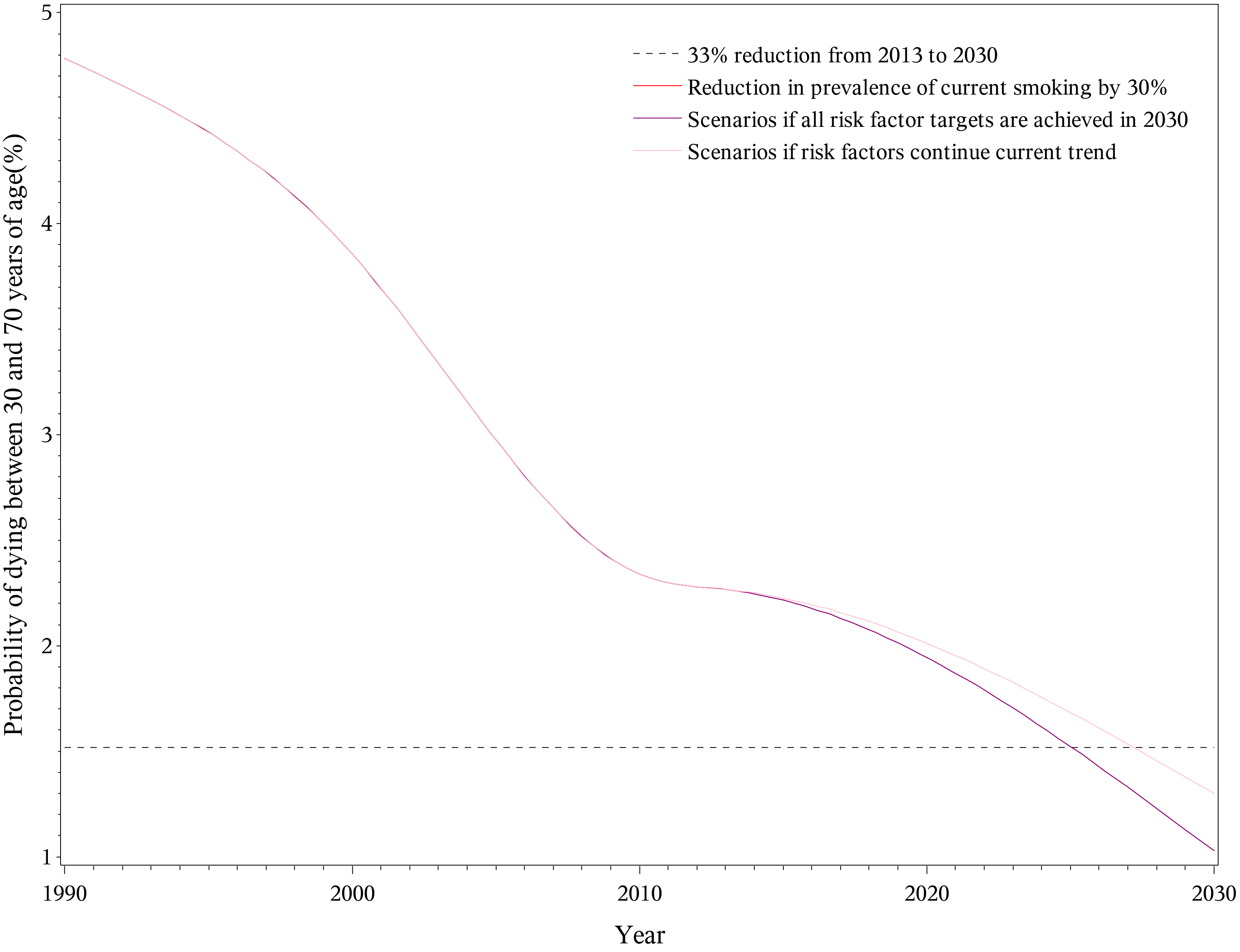

Figure S9

Probability of premature death due to Chronic respiratory disease between ages 30-70 in China from 1990 to 2030-Female

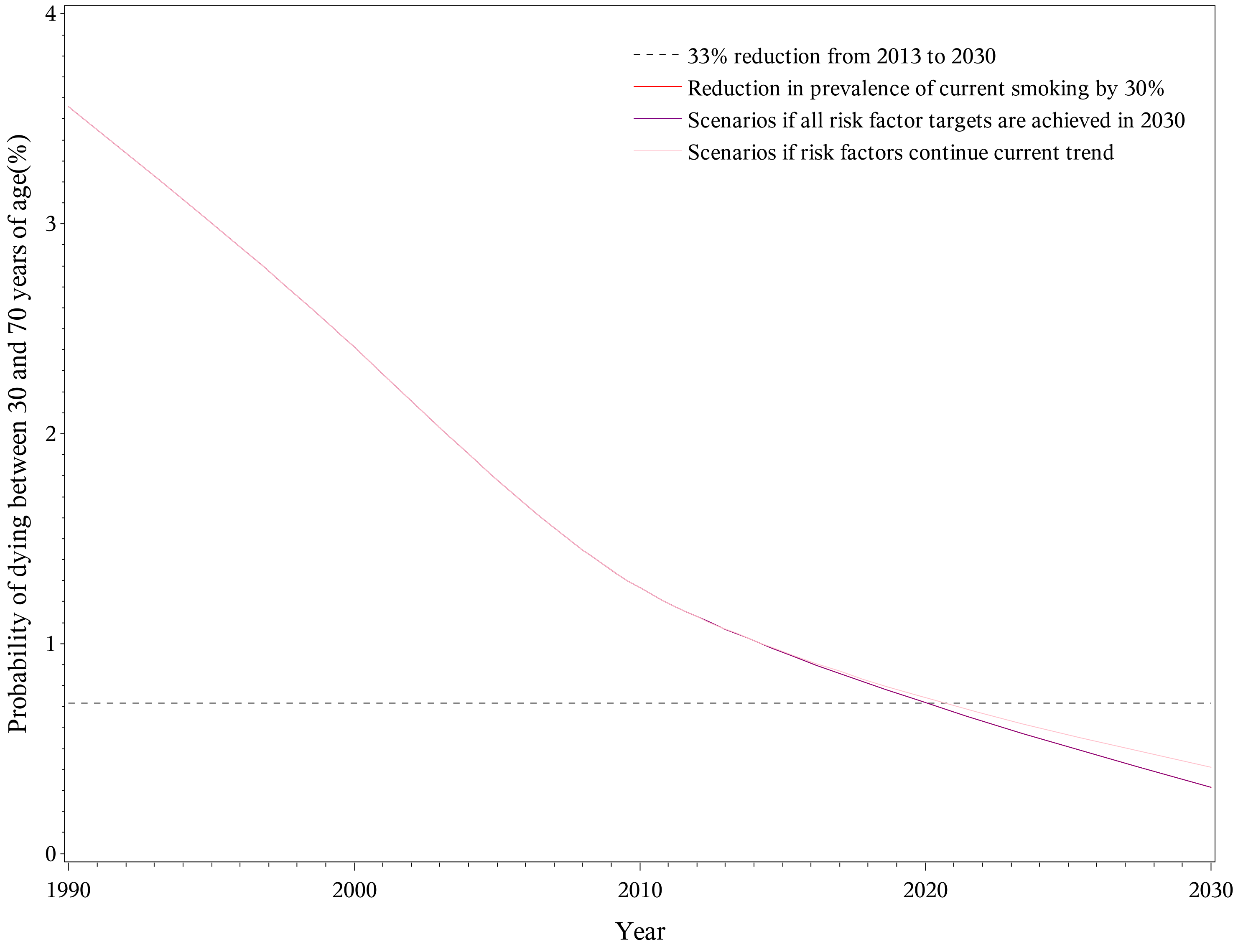

Figure S10

Probability of premature death due to Diabetes mellitus between ages 30-70 in China from 1990 to 2030-Male

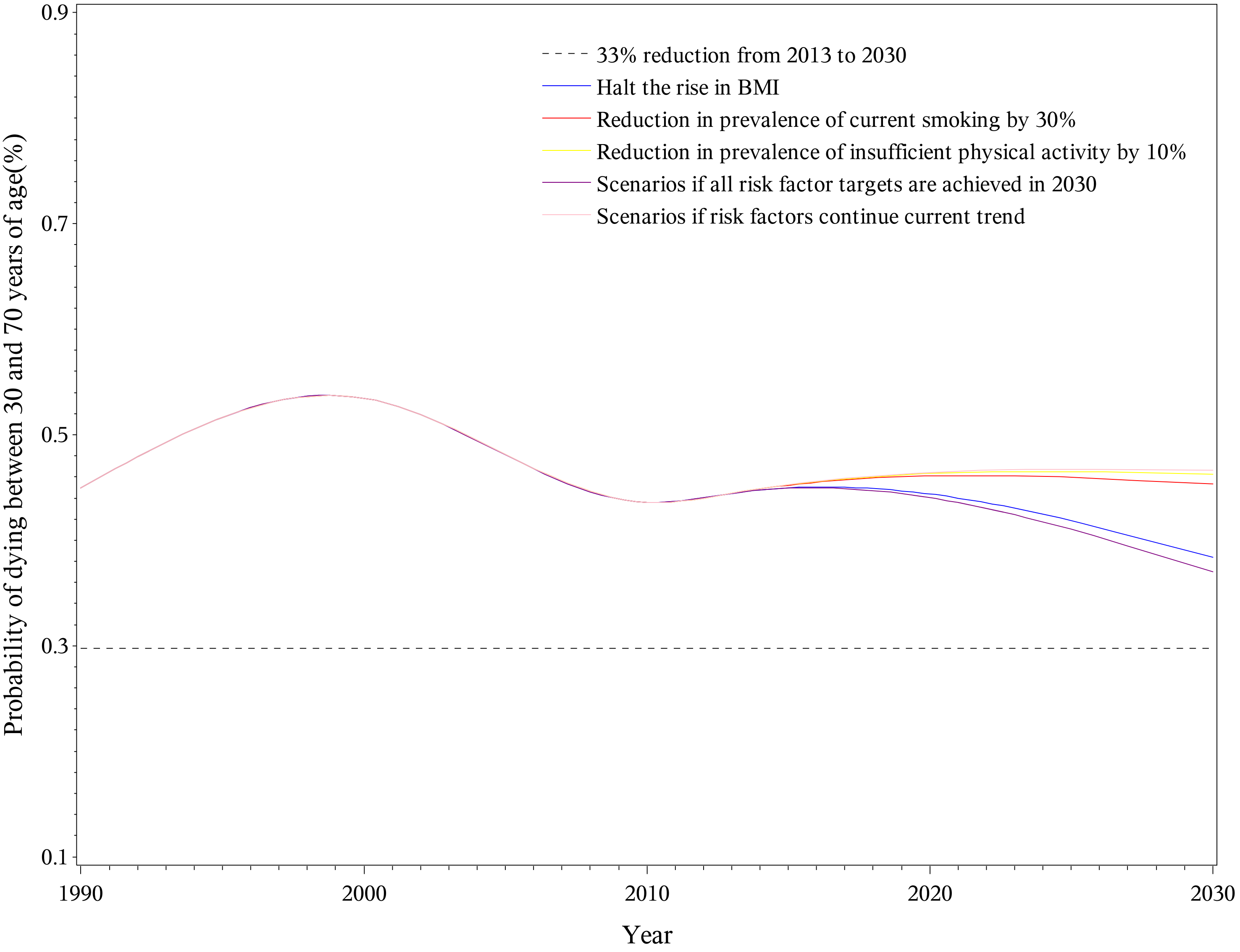

Figure S11

Probability of premature death due to Diabetes mellitus between ages 30-70 in China from 1990 to 2030-Female

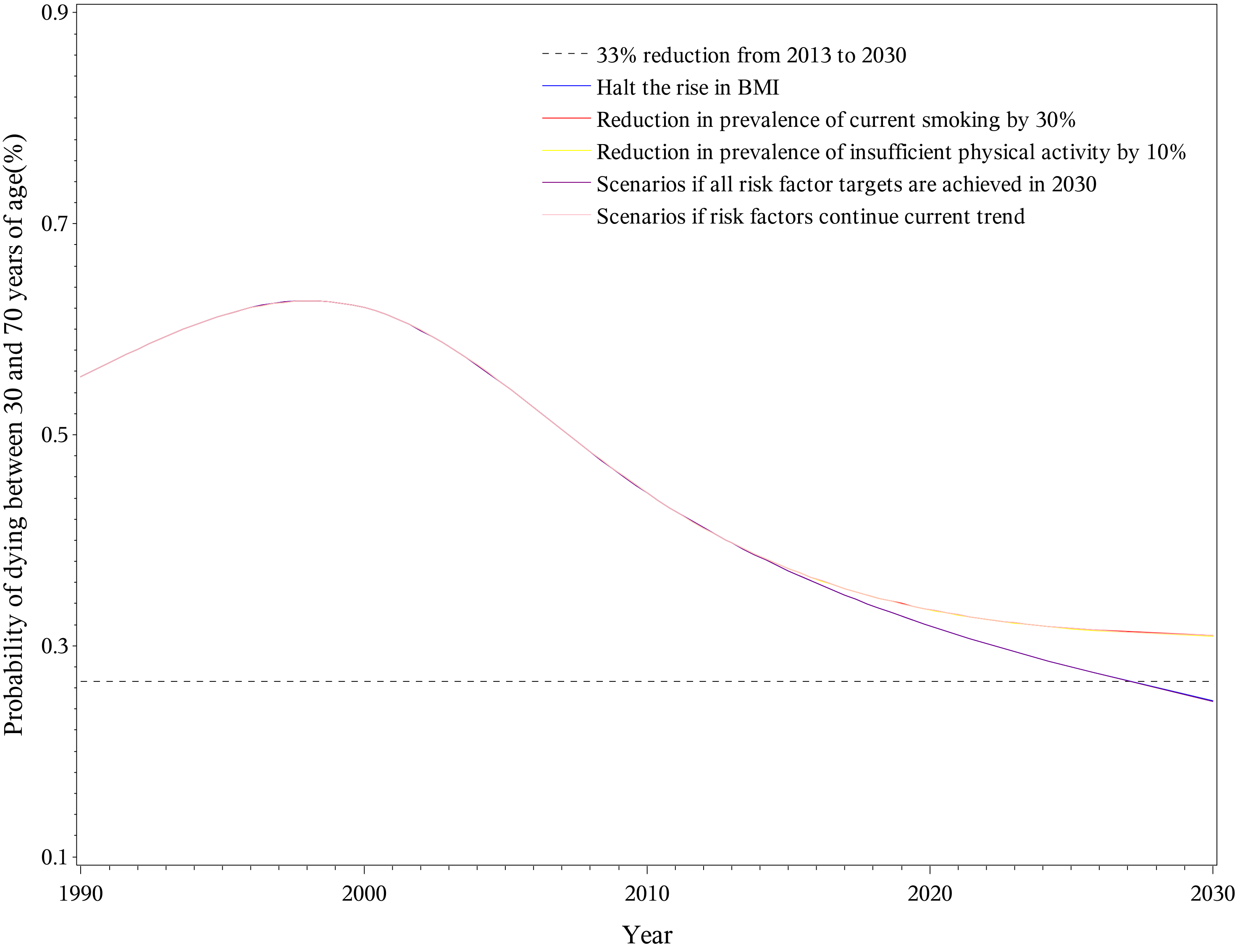

Additional file Tables

Table S1 Deaths and premature mortality of main NCDs for population aged 30 to 70 in 2013 and projections for 2030 if risk factor trends continue

Gender	Disease	2013		2030		% Change	
		Premature deaths (in thousands)	Premature mortality (%)	Premature deaths (in thousands)	Premature mortality if risk factor trends continue (%)	Absolute change in premature deaths	Premature mortality
Both	Total	3108	19.8	3521	17.2	13.3	-13.1
	CVD	1241	8.6	1518	7.8	22.3	-9.3
	Ischemic stroke	174	1.4	276	1.4	58.6	0
	Haemorrhagic stroke	487	3.4	513	2.7	5.3	-20.6
	Ischemic heart disease	450	3.2	639	3.4	42	6.2
	Hypertensive heart disease	58	0.4	50	0.3	-13.8	-25
	Other CVDs	72	0.5	40	0.2	-44.4	-60
	Cancer	1270	8.3	1444	7.5	13.7	-9.6
	Colon and rectum cancer	80	0.6	106	0.6	32.5	0
	Oesophageal cancer	111	0.8	117	0.6	5.4	-25
	Liver cancer	259	1.6	269	1.5	3.9	-6.3
	Tracheal, bronchus, and lung cancer	302	2.2	412	2.2	36.4	0
	Stomach cancer	174	1.3	159	0.9	-8.6	-30.8
	Other cancers	344	2.2	382	2.1	11	-4.5
	Diabetes mellitus	59	0.4	75	0.4	27.1	0
	Chronic respiratory diseases	210	1.7	165	0.9	-21.4	-47.1
	COPD	179	1.4	130	0.7	-27.4	-50
	Other chronic respiratory diseases	32	0.2	35	0.2	9.4	0
	Other NCDs	328	2.1	319	1.7	-2.7	-19
Men	Total	2043	25.4	2453	23.5	20.1	-7.5
	CVD	806	11.2	1041	10.8	29.2	-3.6
	Ischemic stroke	113	1.8	184	2	62.8	11.1
	Haemorrhagic stroke	313	4.5	339	3.6	8.3	-20
	Ischemic heart disease	309	4.3	464	4.9	50.2	14

	Hypertensive heart disease	35	0.5	32	0.4	-8.6	-20
	Other CVDs	36	0.5	23	0.3	-36.1	-40
	Cancer	847	11.2	1026	10.6	21.1	-5.4
	Colon and rectum cancer	47	0.7	66	0.7	40.4	0
	Oesophageal cancer	89	1.3	101	1.1	13.5	-15.4
	Liver cancer	202	2.5	217	2.3	7.4	-8
	Tracheal, bronchus, and lung cancer	220	3.2	320	3.5	45.5	9.4
	Stomach cancer	127	1.9	124	1.4	-2.4	-26.3
	Other cancers	162	2.1	198	2.1	22.2	0
	Diabetes mellitus	30	0.4	43	0.5	43.3	25
	Chronic respiratory diseases	138	2.3	119	1.3	-13.8	-43.5
	COPD	116	1.9	92	1	-20.7	-47.4
	Other chronic respiratory diseases	22	0.3	27	0.3	22.7	0
	Other NCDs	222	2.8	224	2.4	0.9	-14.3
Women	Total	1065	13.4	1068	10.2	0.3	-23.9
	CVD	435	5.9	478	4.6	9.9	-22
	Ischemic stroke	60	0.9	93	0.9	55	0
	Haemorrhagic stroke	174	2.4	174	1.7	0	-29.2
	Ischemic heart disease	141	2	175	1.7	24.1	-15
	Hypertensive heart disease	23	0.3	18	0.2	-21.7	-33.3
	Other CVDs	36	0.5	17	0.2	-52.8	-60
	Cancer	422	5.3	418	4.3	-0.9	-18.9
	Colon and rectum cancer	33	0.4	39	0.4	18.2	0
	Oesophageal cancer	22	0.3	16	0.2	-27.3	-33.3
	Liver cancer	56	0.7	52	0.5	-7.1	-28.6
	Tracheal, bronchus, and lung cancer	82	1.1	92	0.9	12.2	-18.2
	Stomach cancer	47	0.6	34	0.4	-27.7	-33.3
	Other cancers	183	2.2	184	2	0.5	-9.1
	Diabetes mellitus	28	0.4	32	0.3	14.3	-25
	Chronic respiratory diseases	72	1.1	45	0.4	-37.5	-63.6
	COPD	62	0.9	37	0.3	-40.3	-66.7
	Other chronic respiratory diseases	10	0.1	8	0.1	-20	0
	Other NCDs	108	1.4	95	1	-12	-28.6

Table S2 Reduction in deaths of NCDs for population aged 30 to 70 in 2030 with different risk factor scenarios (in thousands).

		Smoking	Physical activity	High BMI	Fasting glucose	Total cholesterol	SBP	All targets achieved
Both	Total	326.0	7.8	95.6	57.4	52.7	564.1	998.8
	CVD	66.6	6.7	54.9	57.4	52.7	564.1	703.1
	Ischaemic stroke	10.7	2.2	5.6	9.3	7.6	114.4	133.8
	Haemorrhagic stroke	21.8	0	14.3	19.1	0.0	228.3	256.9
	Ischaemic heart disease	32.2	4.5	27.0	29.0	45.1	221.3	302.8
	Hypertensive heart disease	2.0	0	8.0	0	0	0	9.7
	Other CVD	-	-	-	-	-	-	-
	Cancer	222.4	0.6	26.9	0	0	0	244.8
	Colon and rectum cancer	8.3	0.6	3.1	0	0	0	11.7
	Oesophageal cancer	29.6	0	8.3	0	0	0	35.8
	Liver cancer	45.6	0	15.5	0	0	0	58.5
	Tracheal, bronchus, and lung cancer	117.5	0	0	0	0	0	117.5
	Stomach cancer	21.4	0	0	0	0	0	21.4
	Other Caner	-	-	-	-	-	-	-
	Diabetes mellitus	1.2	0.4	13.8	0	0	0	15.1
	Chronic respiratory diseases	35.8	0	0	0	0	0	35.8
	COPD	35.8	0	0	0	0	0	35.8
	Other Chronic respiratory diseases	-	-	-	-	-	-	-
	Other NCDs	-	-	-	-	-	-	-
Men	Total	280.1	7.1	67.4	39.2	37.3	391.9	734.8
	CVD	75.0	6.2	37.1	39.2	37.3	391.9	503.3
	Ischaemic stroke	12.4	2.0	3.5	6.0	4.9	76.7	92.3
	Haemorrhagic stroke	25.0	0.0	9.3	12.4	0	152.8	176.7
	Ischaemic heart disease	35.5	4.1	19.4	20.8	32.4	162.4	227.7
	Hypertensive heart disease	2.2	0	4.9	0	0	0	6.7
	Other CVD	-	-	-	-	-	-	-
	Cancer	178.7	0.5	22.8	0	0	0	197.6
	Colon and rectum cancer	4.8	0.5	2.5	0	0	0	7.6
	Oesophageal cancer	25.6	0	7.0	0	0	0	30.9

	Liver cancer	39.0	0	13.3	0	0	0	49.9
	Tracheal, bronchus, and lung cancer	91.6	0	0	0	0	0	91.6
	Stomach cancer	17.7	0	0	0	0	0	17.7
	Other Caner	-	-	-	-	-	-	-
	Diabetes mellitus	1.2	0.4	7.5	0	0	0	8.8
	Chronic respiratory diseases	25.2	0	0	0	0	0	25.2
	COPD	25.2	0	0	0	0	0	25.2
	Other Chronic respiratory diseases	-	-	-	-	-	-	-
	Other NCDs	-	-	-	-	-	-	-
Women	Total	45.9	0.7	28.2	18.1	15.4	172.2	264.0
	CVD	-8.4	0.6	17.8	18.1	15.4	172.2	199.8
	Ischaemic stroke	-1.7	0.2	2.0	3.3	2.7	37.7	41.6
	Haemorrhagic stroke	-3.2	0	5.0	6.6	0	75.5	80.2
	Ischaemic heart disease	-3.3	0.4	7.6	8.2	12.7	58.9	75.1
	Hypertensive heart disease	-0.2	0	3.1	0	0	0	3.0
	Other CVD	-	-	-	-	-	-	-
	Cancer	43.8	0.1	4.1	0	0	0	47.3
	Colon and rectum cancer	3.5	0.1	0.6	0	0	0	4.1
	Oesophageal cancer	4.0	0	1.2	0	0	0	4.9
	Liver cancer	6.6	0	2.3	0	0	0	8.6
	Tracheal, bronchus, and lung cancer	25.9	0	0	0	0	0	25.9
	Stomach cancer	3.8	0	0	0	0	0	3.8
	Other Caner	-	-	-	-	-	-	-
	Diabetes mellitus	0	0.1	6.3	0	0	0	6.3
	Chronic respiratory diseases	10.6	0	0	0	0	0	10.6
	COPD	10.6	0	0	0	0	0	10.6
	Other Chronic respiratory diseases	-	-	-	-	-	-	-
	Other NCDs	-	-	-	-	-	-	-

Table S3 NCDs deaths (in thousands) in 2030 for population of all ages if risk factor trends continue

		2013	2030	% change
Both	Total	849.9	1216.1	43.1
	CVD	401.7	639.8	59.3
	Ischemic stroke	79.6	161.8	103.3
	Haemorrhagic stroke	127.7	155.7	21.9
	Ischemic heart disease	150.1	277.7	85.0
	Hypertensive heart disease	25.9	31.3	20.8
	Other CVD	18.4	13.3	-27.7
	Cancer	226.1	308.4	36.4
	Colon and rectum cancer	16	26	62.5
	Oesophageal cancer	20.9	25.5	22.0
	Liver cancer	38.1	46.6	22.3
	Tracheal, bronchus, and lung cancer	58.1	97.2	67.3
	Stomach cancer	33.8	37.1	9.8
	Other Caner	59.2	76	28.4
	Diabetes mellitus	14.4	25.3	75.7
	Chronic respiratory diseases	115.2	123.3	7.0
	COPD	98.1	93.1	-5.1
	Other Chronic respiratory diseases	17.1	30.2	76.6
	Other NCDs	92.5	119.3	29.0
Men	Total	487.1	695.6	42.8
	CVD	218.1	346.4	58.8
	Ischemic stroke	42.8	81.5	90.4
	Haemorrhagic stroke	71.7	85.5	19.2
	Ischemic heart disease	82.4	157.7	91.4
	Hypertensive heart disease	12.6	15.2	20.6
	Other CVD	8.7	6.5	-25.3
	Cancer	145.5	201.7	38.6
	Colon and rectum cancer	8.9	14.4	61.8
	Oesophageal cancer	15.6	20.2	29.5
	Liver cancer	27.8	33.5	20.5
	Tracheal, bronchus, and lung cancer	40.7	68.8	69.0
	Stomach cancer	23.3	25.9	11.2
	Other Caner	29.3	38.9	32.8
	Diabetes mellitus	6.7	11.6	73.1
	Chronic respiratory diseases	64.2	71.5	11.4
	COPD	54.9	54	-1.6
	Other Chronic respiratory diseases	9.3	17.5	88.2
	Other NCDs	52.6	64.4	22.4
Women	Total	362.8	520.5	43.5

CVD	183.6	293.4	59.8
Ischemic stroke	36.9	80.2	117.3
Haemorrhagic stroke	55.9	70.3	25.8
Ischemic heart disease	67.7	120	77.3
Hypertensive heart disease	13.3	16.1	21.1
Other CVD	9.7	6.8	-29.9
Cancer	80.6	106.6	32.3
Colon and rectum cancer	7.1	11.6	63.4
Oesophageal cancer	5.3	5.3	0.0
Liver cancer	10.3	13.1	27.2
Tracheal, bronchus, and lung cancer	17.4	28.4	63.2
Stomach cancer	10.5	11.2	6.7
Other Caner	30	37.1	23.7
Diabetes mellitus	7.6	13.7	80.3
Chronic respiratory diseases	51	51.8	1.6
COPD	43.3	39.1	-9.7
Other Chronic respiratory diseases	7.7	12.7	64.9
Other NCDs	40	55	37.5

Table S4 Comparison of projected deaths and premature mortality of main NCDs in 2030 for population aged 30-69 between the scenarios in which risk factors continue past trend and the method of proportional change model.

		Deaths (in thousands)		Premature mortality (%)	
		if risk factor trends continue	Proportional Change model	if risk factor trends continue	Proportional Change model
Both	Total	3521	3336	17.2	16.4
	CVD	1518	1331	7.8	6.8
	Ischemic stroke	276	241	1.4	1.3
	Haemorrhagic stroke	513	459	2.7	2.4
	Ischemic heart disease	639	542	3.4	2.9
	Hypertensive heart disease	50	48	0.3	0.3
	Other CVD	40	40	0.2	0.2
	Cancer	1444	1446	7.5	7.6
	Colon and rectum cancer	106	106	0.6	0.6
	Oesophageal cancer	117	117	0.6	0.6
	Liver cancer	269	270	1.5	1.5
	Tracheal, bronchus, and lung cancer	412	413	2.2	2.2
	Stomach cancer	159	159	0.9	0.9
	Other Caner	382	382	2.1	2.1
	Diabetes mellitus	75	75	0.4	0.4
	Chronic respiratory diseases	165	165	0.9	0.9
	COPD	130	130	0.7	0.7
	Other Chronic respiratory diseases	35	35	0.2	0.2
	Other NCDs	319	319	1.7	1.7
				0	0.0
Men	Total	2453	2354	23.5	22.7
	CVD	1041	941	10.8	9.8
	Ischemic stroke	184	166	2	1.8
	Haemorrhagic stroke	339	314	3.6	3.4
	Ischemic heart disease	464	406	4.9	4.4
	Hypertensive heart disease	32	32	0.4	0.4

	Other CVD	23	23	0.3	0.3
	Cancer	1026	1027	10.6	10.6
	Colon and rectum cancer	66	66	0.7	0.7
	Oesophageal cancer	101	101	1.1	1.1
	Liver cancer	217	217	2.3	2.3
	Tracheal, bronchus, and lung cancer	320	320	3.5	3.5
	Stomach cancer	124	124	1.4	1.4
	Other Caner	198	198	2.1	2.1
	Diabetes mellitus	43	43	0.5	0.5
	Chronic respiratory diseases	119	119	1.3	1.3
	COPD	92	92	1	1.0
	Other Chronic respiratory diseases	27	27	0.3	0.3
	Other NCDs	224	224	2.4	2.4
				0	0.0
Female	Total	1068	983	10.2	9.4
	CVD	478	390	4.6	3.7
	Ischemic stroke	93	75	0.9	0.7
	Haemorrhagic stroke	174	145	1.7	1.4
	Ischemic heart disease	175	136	1.7	1.3
	Hypertensive heart disease	18	17	0.2	0.2
	Other CVD	17	17	0.2	0.2
	Cancer	418	420	4.3	4.3
	Colon and rectum cancer	39	39	0.4	0.4
	Oesophageal cancer	16	16	0.2	0.2
	Liver cancer	52	52	0.5	0.5
	Tracheal, bronchus, and lung cancer	92	93	0.9	0.9
	Stomach cancer	34	35	0.4	0.4
	Other Caner	184	184	2	2.0
	Diabetes mellitus	32	32	0.3	0.3
	Chronic respiratory diseases	45	45	0.4	0.4
	COPD	37	38	0.3	0.3
	Other Chronic respiratory diseases	8	8	0.1	0.1
	Other NCDs	95	95	1	1.0

Table S5 Means and SD of systolic blood pressure among Chinese population by age and sex in 1990, 2013, and 2030*.

Age	Male, mean (SD) – mm Hg			Female, mean (SD) – mm Hg		
	1990	2013	2030*	1990	2013	2030*
25-	110.6(10.5)	124.0(14.0)	135.0(17.3)	105.0(9.7)	114.8(14.4)	122.6(19.2)
30-	115.5(13.1)	124.9(14.8)	132.4(16.2)	108.5(11.9)	116.2(14.8)	122.2(17.4)
35-	115.1(13.1)	126.8(15.9)	136.1(18.5)	107.4(11.8)	118.7(15.8)	127.8(19.7)
40-	119.3(13.2)	127.7(16.5)	134.3(19.5)	115.6(12.5)	122.8(17.3)	128.3(22.0)
45-	119.4(13.0)	130.2(17.9)	138.9(22.7)	116.1(12.5)	127.8(19.2)	137.2(26.4)
50-	124.7(13.5)	132.9(18.9)	139.3(24.3)	125.5(13.7)	132.4(20.6)	137.6(27.9)
55-	127.3(13.7)	135.6(19.9)	142.1(26.2)	127.2(13.7)	135.2(21.0)	141.5(28.8)
60-	133.7(14.4)	138.0(20.4)	141.2(26.4)	134.5(14.5)	138.8(21.8)	142.1(29.4)
65-	132.0(13.8)	141.1(21.4)	148.2(29.6)	135.6(14.4)	142.6(22.4)	148.0(31.1)
70-	138.7(15.0)	142.9(21.6)	146.1(28.3)	140.1(15.3)	144.5(22.7)	147.9(30.6)
75-	137.5(14.2)	143.1(21.7)	147.4(29.6)	143.0(15.1)	145.4(23.4)	147.2(32.3)
80+	140.3(15.1)	143.8(22.1)	146.5(29.4)	144.9(15.8)	145.7(24.0)	146.4(32.7)

* Projection based on the assumption that past trends between 1990 and 2013 would continue to 2030.

Table S6 Means and SD of body mass index among Chinese population by age and sex in 1990, 2013, and 2030.

Age	Male, mean (SD) – kg/m^2			Female, mean (SD) – kg/m^2		
	1990	2013	2030*	1990	2013	2030*
25-	20.9(2.7)	23.0(3.3)	24.7(3.9)	21.3(2.7)	21.9(3.3)	22.3(3.9)
30-	21.6(2.8)	23.5(3.2)	25.0(3.6)	21.9(2.7)	22.7(3.3)	23.1(3.8)
35-	21.6(2.7)	23.8(3.2)	25.5(3.5)	21.9(2.8)	23.3(3.3)	24.2(3.9)
40-	21.8(2.9)	24.0(3.1)	25.8(3.2)	22.5(2.9)	23.8(3.2)	24.8(3.4)
45-	21.8(3.0)	24.3(3.0)	26.1(2.9)	22.3(3.1)	24.3(3.3)	25.9(3.4)
50-	21.9(3.1)	23.9(3.0)	25.5(2.9)	22.4(3.4)	24.4(3.3)	25.9(3.2)
55-	22.4(3.1)	23.8(2.9)	24.8(2.8)	22.5(3.8)	24.4(3.5)	25.9(3.2)
60-	22.1(3.0)	23.6(3.1)	24.6(3.1)	22.5(3.6)	24.3(3.6)	25.6(3.5)
65-	22.2(3.1)	23.5(3.1)	24.4(3.1)	22.2(3.6)	24.1(3.8)	25.5(3.9)
70-	21.5(3.2)	23.2(3.2)	24.6(3.2)	22.0(3.2)	23.9(4.1)	25.5(4.8)
75-	21.0(3.2)	22.7(3.6)	24.1(3.9)	20.6(3.7)	23.2(4.1)	25.5(4.4)
80+	20.8(3.4)	22.0(3.6)	23.3(3.7)	21.1(3.2)	22.3(3.8)	23.4(4.3)

* Projection based on the assumption that past trends between 1990 and 2013 would continue to 2030.

Table S7 Means and SD of total cholesterol among Chinese population by age and sex in 1990, 2013, and 2030.

Age	Male, mean (SD) – mmol/L			Female, mean (SD) –mmol/L		
	1990	2013	2030*	1990	2013	2030*
25-	3.8(0.9)	3.9(1.0)	3.9(1.0)	3.8(0.9)	3.7(0.9)	3.7(0.8)
30-	4.2(1.1)	4.1(1.0)	4.1(1.0)	4.0(1.0)	3.8(0.9)	3.8(0.8)
35-	4.3(1.1)	4.2(1.0)	4.2(1.0)	4.1(1.0)	3.8(0.9)	3.8(0.9)
40-	4.4(1.1)	4.2(1.1)	4.2(1.0)	4.3(1.1)	4.0(1.0)	4.0(0.9)
45-	4.4(1.2)	4.3(1.1)	4.3(1.1)	4.5(1.2)	4.1(1.0)	4.1(0.9)
50-	4.6(1.2)	4.3(1.1)	4.3(1.0)	4.7(1.3)	4.4(1.1)	4.4(1.0)
55-	4.5(1.2)	4.2(1.1)	4.2(1.0)	4.9(1.3)	4.4(1.2)	4.4(1.0)
60-	4.7(1.2)	4.2(1.1)	4.2(0.9)	5.0(1.4)	4.5(1.2)	4.5(1.0)
65-	4.3(1.1)	4.2(1.1)	4.2(1.0)	4.8(1.3)	4.5(1.2)	4.5(1.1)
70-	4.5(1.2)	4.2(1.0)	4.2(1.0)	5.0(1.4)	4.5(1.2)	4.5(1.0)
75-	4.1(1.0)	4.3(1.1)	4.3(1.1)	4.5(1.2)	4.6(1.2)	4.6(1.2)
80+	4.2(1.0)	4.1(1.0)	4.1(1.0)	4.8(1.3)	4.4(1.1)	4.4(1.1)

* Projection based on the assumption that past trends between 1990 and 2013 would continue to 2030.

Table S8 Means and SD of fasting plasma glucose among Chinese population by age and sex in 1990, 2013, and 2030.

Age	Male, mean (SD) – mmol/L			Female, mean (SD) – mmol/L		
	1990	2013	2030*	1990	2013	2030*
25-	5.0(1.2)	5.3(1.3)	5.5(1.4)	4.9(1.2)	5.1(1.3)	5.3(1.3)
30-	5.0(1.2)	5.4(1.4)	5.7(1.5)	4.9(1.2)	5.2(1.3)	5.5(1.4)
35-	5.2(1.3)	5.5(1.4)	5.7(1.5)	5.1(1.3)	5.3(1.3)	5.4(1.4)
40-	5.2(1.3)	5.7(1.5)	6.1(1.6)	5.0(1.2)	5.4(1.4)	5.8(1.5)
45-	5.4(1.4)	5.8(1.5)	6.1(1.7)	5.2(1.3)	5.5(1.4)	5.8(1.5)
50-	5.4(1.4)	5.9(1.6)	6.2(1.7)	5.2(1.3)	5.7(1.5)	6.1(1.7)
55-	5.5(1.4)	5.8(1.5)	6.0(1.6)	5.5(1.4)	5.8(1.5)	6.0(1.6)
60-	5.5(1.4)	5.9(1.6)	6.1(1.7)	5.6(1.4)	5.9(1.6)	6.1(1.7)
65-	5.6(1.5)	5.8(1.6)	6.0(1.6)	5.6(1.5)	5.9(1.6)	6.1(1.7)
70-	5.7(1.5)	5.8(1.5)	5.8(1.5)	5.6(1.4)	5.9(1.6)	6.3(1.7)
75-	5.6(1.5)	5.8(1.6)	6.1(1.6)	5.7(1.5)	5.7(1.5)	5.8(1.5)
80+	5.5(1.4)	5.6(1.5)	5.6(1.5)	5.4(1.4)	5.9(1.6)	6.2(1.7)

* Projection based on the assumption that past trends between 1990 and 2013 would continue to 2030.

Table S9 Distribution of physical activity levels among Chinese population by age and sex in 1990, 2013, and 2030.

Age	Physical activity level (METs·min/week)	Male – %			Female – %		
		1990	2013	2030*	1990	2013	2030*
25-	<600	19.8	21.6	23.6	9.4	11.0	12.3
	600-3,999	32.3	30.7	29.3	34.5	34.4	34.3
	4,000-7,999	18.7	18.7	18.6	17.8	16.5	15.5
	>=8,000	29.2	29.0	28.5	38.3	38.1	37.9
30-	<600	20.6	21.1	22.2	7.9	9.1	10.0
	600-3,999	30.8	29.5	28.4	34.2	34.1	34.0
	4,000-7,999	18.1	18.1	17.9	17.8	17.0	16.5
	>=8,000	30.5	31.3	31.6	40.1	39.8	39.5
35-	<600	20.2	20.5	21.5	7.0	7.9	8.6
	600-3,999	29.7	28.2	26.7	33.5	32.5	31.8
	4,000-7,999	18.0	18.0	17.8	17.6	17.3	17.0
	>=8,000	32.1	33.3	34.0	41.9	42.3	42.7
40-	<600	18.6	19.6	21.3	6.8	7.4	8.0
	600-3,999	29.2	26.6	24.5	32.6	29.8	27.8
	4,000-7,999	18.3	18.5	18.4	17.4	17.2	17.0
	>=8,000	33.9	35.3	35.8	43.2	45.6	47.2
45-	<600	17.2	18.4	20.3	6.8	7.2	7.6
	600-3,999	30.3	27.3	24.9	33.6	30.3	27.9
	4,000-7,999	18.5	18.8	18.7	17.8	17.6	17.4
	>=8,000	34.0	35.5	36.1	41.8	44.9	47.1
50-	<600	16.0	17.0	18.4	7.1	7.3	7.4
	600-3,999	33.4	30.4	28.1	36.3	33.8	31.9
	4,000-7,999	18.6	18.8	18.7	18.9	18.4	18.1
	>=8,000	32.0	33.8	34.8	37.7	40.5	42.6
55-	<600	16.3	18.1	19.8	8.0	8.3	8.2
	600-3,999	37.8	34.6	32.2	40.2	37.6	35.7
	4,000-7,999	18.4	18.5	18.5	19.3	18.9	18.5
	>=8,000	27.5	28.8	29.5	32.5	35.2	37.6
60-	<600	18.1	21.6	24.6	9.7	10.1	9.9
	600-3,999	43.9	40.2	37.3	45.6	42.1	39.6
	4,000-7,999	18.0	18.1	18.0	19.0	19.0	18.9
	>=8,000	20.0	20.1	20.1	25.7	28.8	31.6
65-	<600	20.3	24.9	28.8	11.6	12.2	12.1
	600-3,999	46.8	42.6	39.4	48.4	44.5	41.8
	4,000-7,999	17.4	17.3	17.1	18.6	18.7	18.8
	>=8,000	15.5	15.2	14.7	21.4	24.6	27.3
70-	<600	22.6	28.0	32.5	13.7	14.7	15.0
	600-3,999	46.2	41.6	38.0	48.3	44.3	41.6
	4,000-7,999	16.6	16.4	16.0	17.6	17.6	17.6
	>=8,000	14.6	14.0	13.5	20.4	23.4	25.8
75-	<600	25.1	31.0	36.2	15.8	17.2	18.0
	600-3,999	45.5	40.6	36.6	48.1	44.2	41.5
	4,000-7,999	15.8	15.4	14.9	16.7	16.5	16.4
	>=8,000	13.6	13.0	12.3	19.4	22.1	24.1
80+	<600	31.5	39.0	45.7	20.8	23.6	25.7
	600-3,999	43.8	37.9	33.1	48.5	44.7	41.8
	4,000-7,999	13.6	12.9	12.0	14.2	13.4	12.9
	>=8,000	11.1	10.2	9.2	16.5	18.3	19.6

* Projection based on the assumption that past trends between 1990 and 2013 would continue to 2030.

Table S10 Smoking prevalence among Chinese population by age and sex in 1990, 2013, and 2030.

Age	Male – %			Female – %		
	1990	2013	2030*	1990	2013	2030*
30-	61.2	44.0	34.9	1.9	0.7	0.4
35-	65.1	47.7	38.0	2.8	1.0	0.5
40-	64.8	51.6	45.7	4.3	1.4	0.6
45-	65.6	55.4	49.9	6.8	2.1	0.7
50-	66.5	56.9	50.0	9.6	2.5	0.9
55-	65.5	55.7	49.0	11.3	2.6	0.9
60-	63.3	52.3	44.8	13.4	3.0	1.0
65-	61.4	46.8	38.1	15.3	3.6	1.6
70-	59.2	41.0	30.0	15.1	5.2	2.4
75-	52.7	35.4	26.4	13.9	6.0	3.8
80+	51.1	33.2	24.8	12.1	5.6	3.1

* Projection based on the assumption that past trends between 1990 and 2013 would continue to 2030.
